# Integrative linkage mapping, GWAS, and RNA-Seq analysis unravel the genetic architecture and candidate genes for drought tolerance in *Chrysanthemum* interspecific F_1_ progeny

**DOI:** 10.1093/hr/uhaf169

**Published:** 2025-06-25

**Authors:** Zhaowen Lu, Jiangshuo Su, Yu Xiang, Xuefeng Zhang, Shiyun Wen, Zhiqiang Geng, Jiafu Jiang, Zhiyong Guan, Weimin Fang, Fadi Chen, Fei Zhang

**Affiliations:** State Key Laboratory of Crop Genetics & Germplasm Enhancement and Utilization, Key Laboratory of Biology of Ornamental Plants in East China, National Forestry and Grassland Administration, College of Horticulture, Nanjing Agricultural University, No. 1 Weigang, Nanjing 210095, China; Zhongshan Biological Breeding Laboratory, No. 50 Zhongling Street, Nanjing 210014, China; State Key Laboratory of Crop Genetics & Germplasm Enhancement and Utilization, Key Laboratory of Biology of Ornamental Plants in East China, National Forestry and Grassland Administration, College of Horticulture, Nanjing Agricultural University, No. 1 Weigang, Nanjing 210095, China; Zhongshan Biological Breeding Laboratory, No. 50 Zhongling Street, Nanjing 210014, China; State Key Laboratory of Crop Genetics & Germplasm Enhancement and Utilization, Key Laboratory of Biology of Ornamental Plants in East China, National Forestry and Grassland Administration, College of Horticulture, Nanjing Agricultural University, No. 1 Weigang, Nanjing 210095, China; Zhongshan Biological Breeding Laboratory, No. 50 Zhongling Street, Nanjing 210014, China; State Key Laboratory of Crop Genetics & Germplasm Enhancement and Utilization, Key Laboratory of Biology of Ornamental Plants in East China, National Forestry and Grassland Administration, College of Horticulture, Nanjing Agricultural University, No. 1 Weigang, Nanjing 210095, China; Zhongshan Biological Breeding Laboratory, No. 50 Zhongling Street, Nanjing 210014, China; State Key Laboratory of Crop Genetics & Germplasm Enhancement and Utilization, Key Laboratory of Biology of Ornamental Plants in East China, National Forestry and Grassland Administration, College of Horticulture, Nanjing Agricultural University, No. 1 Weigang, Nanjing 210095, China; Zhongshan Biological Breeding Laboratory, No. 50 Zhongling Street, Nanjing 210014, China; State Key Laboratory of Crop Genetics & Germplasm Enhancement and Utilization, Key Laboratory of Biology of Ornamental Plants in East China, National Forestry and Grassland Administration, College of Horticulture, Nanjing Agricultural University, No. 1 Weigang, Nanjing 210095, China; Zhongshan Biological Breeding Laboratory, No. 50 Zhongling Street, Nanjing 210014, China; State Key Laboratory of Crop Genetics & Germplasm Enhancement and Utilization, Key Laboratory of Biology of Ornamental Plants in East China, National Forestry and Grassland Administration, College of Horticulture, Nanjing Agricultural University, No. 1 Weigang, Nanjing 210095, China; Zhongshan Biological Breeding Laboratory, No. 50 Zhongling Street, Nanjing 210014, China; State Key Laboratory of Crop Genetics & Germplasm Enhancement and Utilization, Key Laboratory of Biology of Ornamental Plants in East China, National Forestry and Grassland Administration, College of Horticulture, Nanjing Agricultural University, No. 1 Weigang, Nanjing 210095, China; Zhongshan Biological Breeding Laboratory, No. 50 Zhongling Street, Nanjing 210014, China; State Key Laboratory of Crop Genetics & Germplasm Enhancement and Utilization, Key Laboratory of Biology of Ornamental Plants in East China, National Forestry and Grassland Administration, College of Horticulture, Nanjing Agricultural University, No. 1 Weigang, Nanjing 210095, China; Zhongshan Biological Breeding Laboratory, No. 50 Zhongling Street, Nanjing 210014, China; State Key Laboratory of Crop Genetics & Germplasm Enhancement and Utilization, Key Laboratory of Biology of Ornamental Plants in East China, National Forestry and Grassland Administration, College of Horticulture, Nanjing Agricultural University, No. 1 Weigang, Nanjing 210095, China; Zhongshan Biological Breeding Laboratory, No. 50 Zhongling Street, Nanjing 210014, China; State Key Laboratory of Crop Genetics & Germplasm Enhancement and Utilization, Key Laboratory of Biology of Ornamental Plants in East China, National Forestry and Grassland Administration, College of Horticulture, Nanjing Agricultural University, No. 1 Weigang, Nanjing 210095, China; Zhongshan Biological Breeding Laboratory, No. 50 Zhongling Street, Nanjing 210014, China

## Abstract

Drought stress is a major environmental constraint that severely impacts plant production. However, the genetic basis is primarily misunderstood in chrysanthemum species. The objectives of this study are to examine the genetic variation of drought tolerance in reciprocal F_1_ progenies of *Chrysanthemum dichrum* (drought-tolerant) and *Chrysanthemum nankingense* (drought-sensitive) and identify candidate genes by integrating linkage mapping, genome-wide association study (GWAS), and RNA-seq analysis. The results revealed extensive variation for the investigated traits in response to drought stress and notable genetic divergence in drought tolerance between the reciprocal crosses. This confirms that the hybridization direction influenced drought tolerance phenotypes. A high-resolution genetic map containing 6677 nonredundant bin markers spanning 1859.31 cM across nine linkage groups (LGs), achieving an average marker density of 0.28 cM, was developed with a genotyping-by-sequencing (GBS) approach. The inclusive composite interval mapping (ICIM) detected 89 significant quantitative trait loci (QTLs), and GWAS identified 1360 significant quantitative trait nucleotides (QTNs) in Single_Env, 394 QTNs, and 114 quantitative epistatic interactions (QEIs) in the Multi_Env algorithm, as well as six pairs of epistatic loci (QEs) related to drought tolerance. Besides the additive effects, we observed considerable adverse dominant and epistatic effects for the significant loci, explaining why drought tolerance exhibits negative heterosis in reciprocal crosses. The integration of QTL mapping and GWAS revealed 38 colocalized loci harboring 10 known and 15 novel candidate genes, eight validated through RNA-seq and qRT-PCR analyses. Moreover, we identified elite haplotypes yielding higher drought tolerance within the candidate gene *Cn1062070.* The findings help elucidate the genetic architecture of drought tolerance in chrysanthemum species and provide valuable genetic resources for the development of drought-tolerant cultivars.

## Introduction

Drought is a major abiotic stress factor that affects plant growth, development, yield, and quality [1–3]. Global climate change has exacerbated drought conditions in many regions of the world [4, 5]. Chrysanthemum (*Chrysanthemum morifolium* Ramat.) is one of the world's most important commercial ornamental crops, valued highly for its esthetic and economic significance. Water deficits severely restrict the growth and development of chrysanthemums, leading to slow growth, reduced ornamental quality, and decreased yield [4, 5]. In practical production, alleviating drought stress through artificial irrigation consumes a massive amount of freshwater resources, thus significantly increasing production costs. Therefore, elucidating the genetic basis of drought tolerance will provide a theoretical foundation for breeding resource-efficient and highly stress-resistant chrysanthemum varieties, ensuring reliable plant production.

Drought tolerance in plants is a complex quantitative trait controlled by multiple genes, and it must be studied through indirect selection [6]. Currently, reported genetic studies mainly focus on evaluating secondary constitutive traits related to drought tolerance, such as plant and leaf morphology, root architecture, flowering time, yield traits, and various physiological and biochemical indicators [6, 7]. Drought indices, i.e. drought susceptibility index (DSI) and drought tolerance index (DTI), have proven to be reliable methods for dissecting the complex interrelated traits associated with drought tolerance [8, 9]. Combined linkage mapping and genome-wide association studies (GWAS) have uncovered genetic mechanisms and new alleles that enhance yield stability and drought resistance in various species [10–13]. However, because drought tolerance-related traits are controlled by alleles with minor effects and are highly sensitive to environmental influences, genetic improvement of drought tolerance is still in its early stages.

In chrysanthemums, preliminary progress has been made in phenotypic identification, excellent gene mining, germplasm improvement, and innovation in drought tolerance research. Li *et al*. [14] and Luo *et al*. [15] screened a batch of excellent drought-tolerant germplasms by integrative principal component and membership function methods. Integration of association analysis and RNA-seq has elucidated multiple stress response mechanisms in chrysanthemums, including heat tolerance [16], disease resistance [17], and waterlogging tolerance [18]. Several drought stress-related genes have been characterized, including zinc finger proteins, DREBs, HSPs, GRAS, and MYB transcription factors (TFs), primarily involved in ABA signaling, ROS scavenging, and osmotic adjustment pathways. Examples include *DgZFP3* [19], *BBX19-ABF3* [20], *CmNF-YB8* [21], *CmRH56* [22], *CmBBX22* [23], *CmSCL4*, and *CmR1MYB1* [24], offering valuable insights into the molecular mechanisms involved in chrysanthemum response to drought stress. In addition, some novel germplasms have been created by distant hybridization and transgenic tools [25–28], providing essential breeding materials for future genetic improvement. However, the genetic basis of chrysanthemum drought tolerance remains unclear, and the limited availability of superior allelic variations severely restricts the efficient genetic improvement of drought tolerance.

To reveal the genetic mechanism underlying drought tolerance in chrysanthemums, we developed reciprocal F_1_ segregating populations using two diploid chrysanthemum species with contrasting drought tolerance traits: drought-tolerant *Chrysanthemum dichrum* and drought-sensitive *Chrysanthemum nankingense*. The genetic variation for drought tolerance in the reciprocal progenies was systematically evaluated over three consecutive years. A high-density genetic linkage map was constructed using single nucleotide polymorphism (SNP) markers generated by genotyping-by-sequencing (GBS). By integrating linkage mapping, GWAS, and transcriptomic analysis, we identified elite alleles and key candidate genes significantly associated with drought tolerance. The results of this study provide an in-depth insight into the genetic architecture of chrysanthemum drought tolerance and pave an avenue for molecular breeding strategies aimed at developing new chrysanthemums with improved drought tolerance.

## Results

### Phenotypic performance

**Figure 1:**
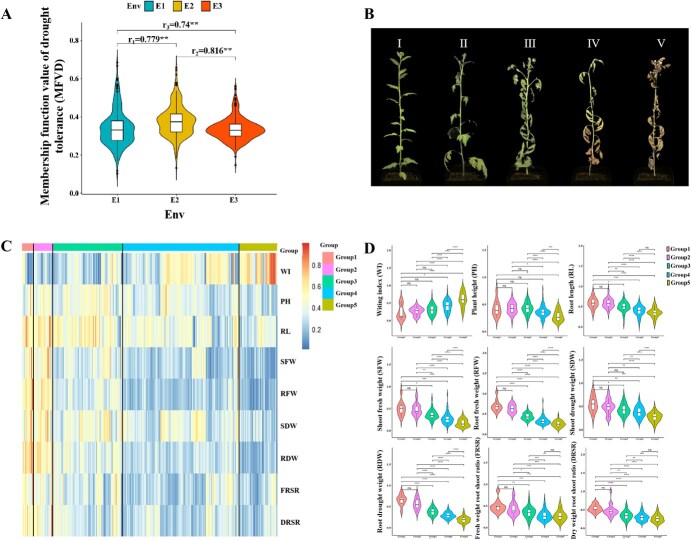
Drought tolerance evaluation and phenotypic characterization in the reciprocal F_1_ progenies of *C. dichrum* and *C. nankingense*. (A) Multiyear correlation analysis of MFVD under three trials (E1, E2, and E3). r_1_, r_2_, and r_3_ refer to the coefficients of Pearson correlation for pairwise comparisons. (B) Representative phenotypes corresponding to five drought tolerance levels (I–V). (C) Heatmap showing nine drought tolerance traits across five genotype groups (Group 1–5) based on drought tolerance classification. (D) Differential analysis of drought tolerance traits among genotype groups. ^*^, ^**^, and ^***^ indicate significance at *P* < 0.05, *P* < 0.01, and *P* < 0.001, respectively.

The original phenotypic data (DC values) are presented in Table S2. All drought tolerance-related traits exhibited extensive and continuous phenotypic variation in the reciprocal interspecific progenies over the 3 years (Fig. S1), with coefficients of variation ranging from 10.65% to 37.31% (Table S3). RFW showed the highest variation under drought stress, followed by WI, SFW, RDW, FRSR, MFVD, and RL. Repeatability analysis of 3-year phenotypic data for 10 drought tolerance traits (Table S4) revealed a repeatability coefficient ranging from 0.69 to 0.85 (*P* < 0.01), indicating high measurement reliability. The MFVD demonstrated prominent year-to-year correlations (Fig. 1A) and significant associations with all drought-related traits (Fig. S2, *P* < 0.01). Based on the BLUE values of MFVD and morphological evaluation criteria (Fig. 1B), we categorized the reciprocal F_1_ hybrids into five drought tolerance groups (Tables S5 and S6). We compared the nine investigated traits across the various groups to explore the key traits resulting in the different drought tolerances (Fig. 1C). The results showed that PH showed no significant intergroup differences, while WI displayed increasing significance from moderate to sensitive groups (*P* < 0.05 ~ *P* < 0.001), and root-related traits (RFW, RDW, DRSR) exhibited the most pronounced differences between resistant (Group 1) and moderate to sensitive groups (Groups 3–5) (*P* < 0.001), suggesting WI and root-related traits as primary indicators for drought response (Fig. 1D).

**Figure 2:**
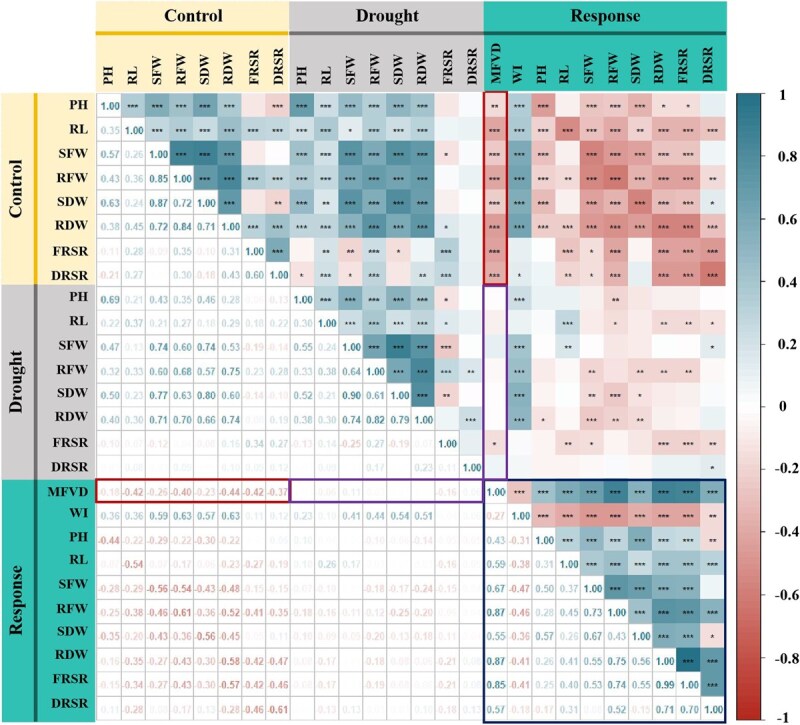
Correlations among the 10 drought tolerance-related traits based on the BLUE values in the reciprocal F_1_ progeny. Control represents the control group (regular watering), Drought represents the experimental group (simulated drought treatment), and Response represents the impact of drought stress on each trait. ^*^ and ^**^ indicate significance at 0.05 and 0.01 levels, respectively.

Our analyses revealed that MFVD was negatively correlated with all investigated traits under control conditions (*P* < 0.01) (Fig. 2, red box) while only maintaining a negative correlation with FRSR under drought stress (purple box, *P* < 0.01). For stress response, MFVD correlated negatively with WI but positively with the other traits (*P* < 0.01), indicating that drought tolerance is driven by stress-induced plasticity rather than constitutive trait values.

### Inheritance of drought tolerance

To dissect the inheritance of drought tolerance in the *Chrysanthemum* reciprocal progenies, we compared the genetic variation, heterosis, and reciprocal effect based on the BLUE values (Table 1). The average performance and coefficient of variation in the reverse cross JHN × YSJ were higher than in the forward cross YSJ × JHN for most investigated traits. The reciprocal crosses differed significantly in the three traits, MFVD, RL, and DRSR. It is worth mentioning that the reciprocal crosses showed negative heterosis for most examined traits. Therefore, drought tolerance generally decayed, though some transgressive segregants in both directions were observed. This suggests that dominant or other nonadditive genetic factors primarily control drought tolerance. To examine parental effects, we found that the reciprocal effects (RE) were positive for most traits, except for WI and PH, where RE < 0. In addition, significant RE was calculated for MFVD, RL, RFW, RDW, FRSR, and DRSR. Thus, the less drought-tolerant JHN is a predominant parent in the reciprocal crosses. In addition, high H^2^ estimates >0.85 were calculated for the drought tolerance-related traits (Table S7).

**Table 1:** Heterosis and reciprocal effects of the 10 drought tolerance-related traits in the reciprocal F_1_ progenies derived from *C. dichrum* (YSJ) and *C. nankingense* (JHN)

Traits	Parents		MPV	*C. dichromum* × *C. nankingense* YSJ × JHN					*C. nankingense* × *C. dichromum* JHN × YSJ					RE
	YSJ	JHN		Mean	SD	*CV* (%)	MPH	Partial parent	Mean	SD	*CV* (%)	MPH	Partial parent	
MFVD	0.49	0.31	0.40	0.32	0.07	21.60	−20.10^**^	JHN	0.37	0.08	20.35	−6.18	JHN	0.06^**^
WI	1.84	3.21	2.52	2.46	0.45	18.42	−2.49	YSJ	2.43	0.78	31.99	−3.89	YSJ	−0.04
PH	0.90	0.79	0.84	0.78	0.08	10.44	−7.00	JHN	0.77	0.08	10.86	−8.71	JHN	−0.01
RL	1.16	0.86	1.01	0.88	0.19	21.69	−12.68^**^	JHN	1.10	0.16	14.52	8.85	YSJ	0.22^**^
SFW	0.81	0.46	0.63	0.46	0.09	19.66	−28.03^**^	JHN	0.47	0.13	26.89	−25.23^**^	JHN	0.02
RFW	1.04	0.48	0.76	0.55	0.17	30.16	−27.43^**^	JHN	0.66	0.23	35.43	−12.85^**^	JHN	0.11^**^
SDW	1.15	0.92	1.03	0.94	0.13	13.98	−9.21^**^	JHN	0.96	0.17	17.18	−6.75	JHN	0.03
RDW	1.75	1.29	1.52	1.45	0.28	18.42	−4.61	JHN	1.54	0.36	23.38	1.32	YSJ	0.09^**^
FRSR	1.15	0.93	1.04	1.32	0.23	22.12	26.92^**^	YSJ	1.44	0.33	22.92	38.46^**^	YSJ	0.12^**^
DRSR	1.67	1.38	1.52	1.44	0.22	15.44	−5.40	JHN	1.63	0.33	20.13	7.14	JHN	0.19^**^

### The high-density genetic linkage map

Utilizing Illumina's HiSeq Xten platform, we conducted successful sequencing of *C. dichrum*, *C. nankingense*, and their reciprocal F_1_ population and obtained 1944.37 Gb of clean data with a clean read ratio close to 100% (Table S8). Statistical analysis confirmed the high sequencing quality (Q20 = 96.35%, Q30 = 93.39%) and consistent GC content ranging from 36.52% to 37.64%. GATK identified a total of 9 643 667 raw SNPs. Considering the characteristics of the CP population, markers of nn × np (22.81%), lm × ll (23.66%), and hk × hk (46.32%) types were selected, and those with missing rates exceeding 15% and showing significant segregation distortion (*P* < 0.001) were excluded from the dataset. Ultimately, we consolidated the filtered 176 647 SNPs into 6677 bin markers, eliminating a marker redundancy of 96.22%, for subsequent linkage mapping.

**Figure 3:**
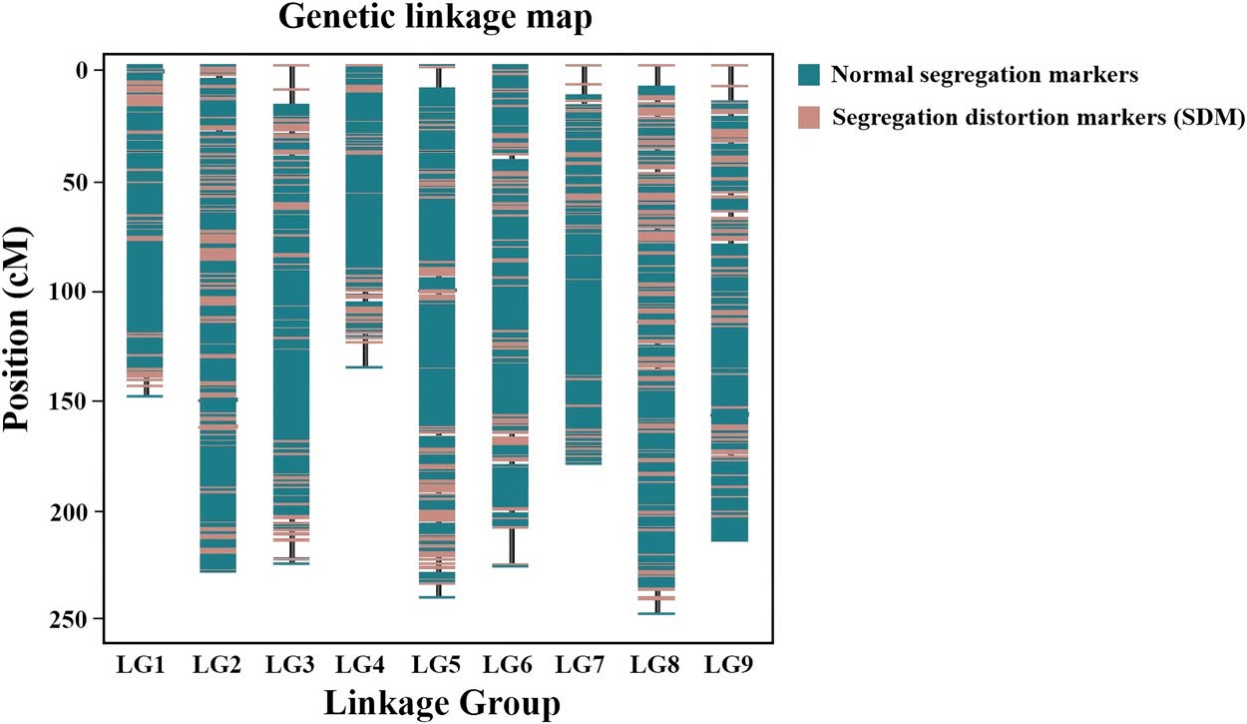
The distribution of SNP markers across the nine linkage groups.

Based on rigorous marker quality control and filtering, we constructed a high-quality genetic map spanning 1859.31 cM for the reciprocal F_1_ population, with an average intermarker distance of 0.28 cM, integrating 6677 bin markers (Fig. 3; Table 2). The lengths of the nine linkage groups vary from 137.4 cM to 249.09 cM, averaging 206.59 cM. The genome-wide recombination rates varied from 0.45 to 1.18 cM/Mb (mean: 0.68 cM/Mb) across chromosomes, with a negative correlation between chromosome length and recombination rate (r = −0.6968, *P* = .0369; Fig. S3). The marker distribution was relatively balanced across chromosomes, with Chr 5 containing the maximum number of markers (883), while Chr 4 had the minimum (506), with an average of 741.89 bin markers per chromosome. Almost all linkage groups (99.82%) feature gaps narrower than 5.0 cM, indicating a high-quality genetic map. Despite the potential minor impact of segregation distortion on mapping accuracy and considering the enhanced coverage of linkage groups, distorted markers were retained within the linkage map [37]. Segregation distortion regions (SDRs) were regions with more than five adjacent loci demonstrating distortion. Notably, 563 bin markers (8.43% of the total) showing significant segregation distortion were successfully integrated into the genetic map. The segregation-distorted markers (SDMs) exhibited uneven distribution across linkage groups, with LG4 (36.76%) and LG8 (20.54%) showing the highest proportions, while LG9 contained no detectable SDMs. A total of 18 SDRs were identified across the genetic maps, primarily located on LG1–LG4, LG6, and LG8, with LG8 and LG3 containing 5 and 4 SDRs respectively, Strong collinearity between the genetic map and physical assembly was confirmed by highly significant Pearson correlations (R = −0.84 to 0.83; *P* = 10^−43^ to 10^−217^) for marker positions across all nine chromosomes (Table S9; Fig. S4).

### Mapping of QTLs for drought tolerance

Based on E1, E2, E3, and BLUE, we detected 89 QTLs significantly associated with the 10 drought tolerance-related traits (Table S10), of which 14 were reproducible QTLs colocated with multiple traits. Individual QTL explained 3.2%–24.41% of the phenotypic variation, with additive effects ranging from −0.25 to 0.24 and dominance effects from −0.39 to 0.28 (Table 3). Drought tolerance in chrysanthemum exhibited predominantly dominant genetic control, with most QTLs (93.26%) showing minor to moderate effects (PVE < 10%). Genetic effect analysis revealed trait-specific inheritance patterns: root traits (RFW, RL) showed predominantly additive effects (RFW: |add| = 0.24 > |dom| = 0.07; RL: |add| = 0.18 > |dom| = 0.07), while MFVD and RDW exhibited comparable additive and dominance effect (MFVD: |add| = 0.04, |dom| = 0.06; RDW: |add| = 0.05, |dom| = 0.05), with dominance effects mainly controlling other traits including WI (|add| = 0.25 < |dom| = 0.56) and SDW (|add| = 0.04 < |dom| = 0.29) (Table 3; Table S10).

In this study, major QTLs were defined by either high phenotypic variation explained (PVE > 10%) or consistent detection across environments. As a result, we figured out 10 major QTLs for drought tolerance, including four significant and six stable QTLs, with additive and dominance effects ranging from −0.25 to 0.24 and − 0.43 to 0.40, respectively (Table 4). These QTLs were distributed across chromosomes 2, 3, 5, 6, 7, and 9, with Chr5 harboring three major QTLs. The pleiotropic QTL qDS-5-19 affected both WI (R^2^ = 15.45%, d/a = 1.56) and SFW (R^2^ = 10.8%, d/a = −0.4), showing overdominance and partial dominance, respectively. qDS-5-22 exhibited the highest PVE (24.41%) with overdominance (d/a = 2.39–3.33) for WI. qDS-9-155 was consistently detected for SDW (E1, E2) with positive effects (0.03–0.06) and overdominance (d/a = 1.67–2.00) and the stable QTL qDS-3-82 for RFW showed consistent partial dominance across E2 and E3 (d/a = 0.8, 0.75, respectively).

### GWAS for drought tolerance

The Single_Env analysis identified 1360 significant QTNs (LOD: 3.09–86.84; PVE: 0.09–23.83%), with additive effects ranging from −0.26 to 0.41 and dominance effects from −0.43 to 0.68 (Table 5; Table S11; Figs S5 and S6). These QTNs were evenly distributed across environments: E1 (332), E2 (351), E3 (340), and BLUE (337), with 115 stable QTNs detected in multiple environments. Chr5 exhibited the highest QTN density, with six loci showing high detection frequency: S1_57209975, S1_6145369, S4_214094985, and S5_232153521 in four environments; S6_355824693 and S6_37834731 in five environments. Among these stable QTNs, 42 were consistent across three environments and 73 across all four, indicating their potential regulatory roles in drought tolerance. The Multi-Env analysis revealed 394 stable QTNs (LOD: 3.02–76.91; PVE: 0.06–9.66%) and 114 environment-specific QEIs (LOD: 3.35–62.87; PVE: 0.16–7.09%), predominantly localized on Chr2 (Table 5; Tables S12 and S13; Fig. S7). Notably, the FRSR-associated QTN S1_247130487 was consistently detected with the highest PVE (23.83%), suggesting its crucial role in drought-responsive root system development. Further analysis revealed 156 QTNs associated with multiple traits, indicating either potential pleiotropic effects or tight linkage of drought-responsive genes. Genetic effect analysis revealed predominant additive control for WI and RL, while other traits showed more substantial dominance effects (Table S14), suggesting dominance-based drought tolerance in the reciprocal population.

The significant QTNs identified through both Single_Env and Multi_Env analyses were distributed across 855 genomic intervals. These intervals showed a size-dependent distribution: 24.12% were small (<100 kb), 48.91% were medium (100 kb–1 Mb), and 27.03% were large (>1 Mb) (Fig. S8A). Notably, medium-sized intervals (100–500 kb) exhibited the maximum phenotypic variance explained (peak R^2^ = 23.83%) and highest proportion of major-effect QTNs (18% with R^2^ > 5%) (Fig. S8B). These results indicated that 100–500 kb represents the optimal resolution range for QTN mapping in our study.

Additionally, we identified six QEs for WI (one for WI2, five for WI3), designated as QE-Chr_Position1 & Chr_Position2 (Table S15). The four QEs, i.e. QE-Chr6_374188342 & Chr7_374188342, QE-Chr1_102006140 & Chr5_10436105, QE-Chr4_201062257 & Chr7_168086983, and QE-Chr5_133363651 & Chr7_317002332 exhibited predominantly additive-by-additive (aa) interactions (aa effects: −0.46 to 0.33). The two vital epistatic interactions were characterized: QE-Chr4_201062257 & Chr7_168086983 showed the highest LOD with significant aa effect (−0.4), displaying antagonistic interaction between its constituent loci (additive effects: −0.03, 0.41); QE-Chr5_133363651 & Chr7_317002332 exhibited the most substantial aa effect (−0.46) and highest PVE (14.57%), showing synergistic interaction.

### Colocalized QTL-GWAS loci for drought tolerance

**Figure 4:**
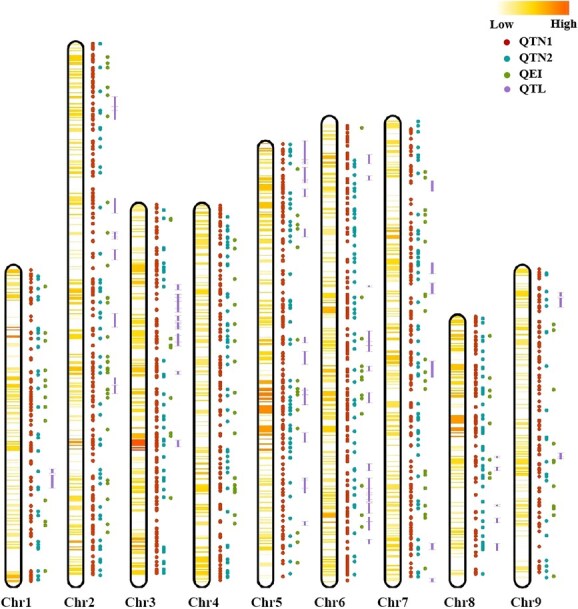
Overview of genomic signatures for drought tolerance detected by GWAS and QTL mapping. The adjacent heatmap illustrates the density of linked SNPs or QTLs across the nine pseudochromosome of *C. nankingense*, calculated as the number of significant loci within a 10 Mb interval. The first column on the right side of the chromosome represents the QTNs identified by GWAS in a Single_Env, and the second column represents the QTNs identified by GWAS in Multi_env. The green dots in the figure represent the QEIs identified by GWAS in Multi_Env.

QTL mapping and GWAS results can be directly cross-validated and complementary. In this study, we identified 26 hotspot regions for drought tolerance within the 10-Mb genomic regions, each containing more than 10 QTLs and QTNs (Fig. 4, Table 6). A major hotspot region was identified on Chr 5 at 190–220 Mb (H5_20–H5_22: 193 562 746–219 164 034 bp), which harbored 4 significant QTLs (purple), 19 single-environment QTNs (red), 7 multienvironment QTNs (blue), and 5 QEIs (green).

Integration of QTL mapping and GWAS identified 31 colocalized QTL-GWAS loci within 23 significant QTLs (Table 7), with the highest density on Chr5 (seven QTLs). The qDS-5-19 region colocalized with three significant SNPs (S5_22774330, S5_28143190, S5_30446876), while six QTLs (qDS-2-34, qDS-3-82, qDS-5-110, qDS-5-164, qDS-6-21, qDS-6-186) overlapped with two SNPs each. Further stringent filtering identified seven consistent significant loci (Table 8). Notably, the S1_247130487 locus, detected in both Sig_Env and Multi_Env analyses, showed the highest PVE (9.66%) and displayed environment-dependent additive effects. This pattern underscores strong genotype-by-environment interaction fluencing fine FRSR under drought stress.

**Table 2:** Basic information of the high-density genetic map of the reciprocal F_1_ population of *C. dichrum* and *C. nankingense*

Chr	Number	Genetic distance (cM)	Average distance (cM)	Max gap (cM)	Gap <5 cM (%)	SDM^a^	SDM (%)	SDR^b^
1	600	150.21	0.25	4.96	100	42	7.00	2
2	851	230.23	0.27	2.2	100	40	4.70	3
3	805	226.11	0.28	10.79	99.63	75	9.32	4
4	506	137.40	0.27	11.75	99.8	186	36.76	3
5	883	241.74	0.27	9.83	99.78	13	1.47	0
6	825	227.57	0.27	17.12	99.88	45	5.46	1
7	712	181.23	0.25	8.32	99.86	10	1.40	0
8	740	249.09	0.33	9.83	99.73	152	20.54	5
9	755	215.73	0.28	8.88	99.74	0	0	0
Mean	741.89	206.59	0.28	9.30	99.82	62.56	9.62	2
Total	6677	1859.31				563		18

a Represents the number of segregation distortion markers.

b Represents the number of segregation distortion regions, and a distorted region with more than five adjacent loci was considered an SDR.

**Table 3:** Summary of 89 significant QTLs associated with the drought tolerance-related traits

Trait	Number	Chr	LOD	R^2^ (%)	add^a^	Total add	dom^b^	Total dom
MFVD	7	2,3,5,6,9	3.41–3.96	5.04–6.33	−0.03–0.02	−0.04	−0.03–0.02	−0.06
WI	13	2,5,6,7,8	3.03–14.8	3.41–24.41	−0.25–0.24	0.25	−0.39–0.24	−0.56
PH	4	2,6,9	3.09–4.4	4.37–6.46	−0.01–0.00	−0.01	−0.24–0.00	−0.18
RL	9	2,3,6,8	3.03–4.9	4.07–6.84	−0.07–0.06	−0.18	−0.14–0.14	−0.07
SFW	10	2,3,5,6	3.13–6.57	4.51–10.8	−0.02–0.05	0.06	−0.05–0.05	−0.13
RFW	12	1,2,3,5,6,9	3.15–8.55	3.9–11.65	−0.13–0.07	−0.24	−0.02–0.08	0.07
SDW	11	3,5,6,7,8,9	3.24–6.81	4.38–9.79	−0.03–0.03	0.04	−0.09–0.00	−0.29
RDW	12	3,5,6,7	3.25–14.19	3.2–8.79	−0.05–0.11	0.05	−0.27–0.28	0.05
FRSR	8	1,2,5,9	3.1–4.22	4.51–6.6	−0.09–0.1	−0.01	−0.12–0.17	0.05
DRSR	3	2,5,8	3.13–3.77	4.61–6.93	−0.06–0.03	−0.10	−0.11–0.16	0.27
Sum	89		3.03–14.8	3.2–24.41	−0.25–0.24	−0.18	−0.39–0.28	−0.85

a represents the additive effect; ^b^ represents the dominance effect.

**Table 4:** The detailed information on major-effect QTLs for drought tolerance-related traits

Trait	Major QTL	Env	Chr	Pos (cM)	Left marker	Right maker	LOD	R^2^ (%)	CI Interval (cM)	Chromosomal region (bp)	add	dom	d/a
WI	qDS-5-19	BLUE	5	19	5_48	5_49	13.43	15.45	18.5–19.5	27337779–29213902	−0.25	−0.39	1.56
	qDS-5-22	E2	5	22	5_59	5_60	14.8	24.41	21.5–22.5	32430115–33234170	−0.18	−0.43	2.39
		E3	5	22	5_59	5_60	11.51	12.72	21.5–22.5	32430115–33234170	−0.12	−0.4	3.33
	qDS-7-180	E1	7	180	7_708	7_709	9.60	10.50	179.5–181	374505745–377945082	0.24	0.02	0.08
RL	qDS-6-105	E2	6	105	6_397	6_398	4.9	6.84	104.5–109.5	173228662–181723241	−0.05	−0.02	0.40
		E3	6	105	6_397	6_398	3.32	5.04	104.5–105.5	173228662–175048927	−0.04	−0.02	0.50
SFW	qDS-5-19	E1	5	19	5_48	5_49	6.57	10.80	16.5–21.5	24657599–32 430 115	0.05	−0.02	−0.40
	qDS-6-184	E1	6	184	6_708	6_709	4.48	7.15	181.5–184.5	300647304–305804727	0.02	−0.01	−0.50
		E3	6	184	6_708	6_709	3.75	5.06	181.5–186.5	300647304–309141884	0.02	−0.01	−0.50
RFW	qDS-3-82	E2	3	82	3_235	3_236	4.32	5.78	80.5–86.5	106950285–115477081	−0.05	−0.04	0.80
		E3	3	82	3_235	3_236	3.72	5.97	80.5–84.5	106950285–112309988	−0.04	−0.03	0.75
	qDS-5-139	BLUE	5	139	5_555	5_556	8.55	11.65	137.5–140.5	201549335–205837618	−0.13	−0.02	0.15
SDW	qDS-9-155	E1	9	155	9_511	9_512	3.34	4.48	153.5–155.5	180315865–183324713	0.03	0.05	1.67
		E2	9	155	9_511	9_512	4.35	6.36	151.5–155.5	178166685–183324713	0.03	0.06	2.00
RDW	qDS-7-58	BLUE	7	58	7_172	7_173	14.19	11.09	57.5–58.5	119994641–122287534	0.00		
DRSR	qDS-2-147	E1	2	147	2_509	2_510	3.77	6.93	145.5–147.5	271436957–276271710	−0.05	0.16	−3.20
		E2	2	147	2_509	2_510	3.23	4.76	145.5–147.5	271436957–276271710	−0.06	0.11	−1.83

**Table 5:** Significant QTNs, QEI, and QEs for drought tolerance detected by the Single_Env and Multi_Env algorithms

Algorithm	QTL type	Number	LOD	R^2^ (%)	add^a^	dom^b^
Single_Env	QTN	1360	3.09–86.84	0.09–23.83	−0.26–0.41	−0.43–0.68
Multi_Env	QTN	394	3.02–76.91	0.06–9.66	−0.21–0.18	−0.19–0.36
	QEI	144	3.35–62.87	0.16–7.09	−0.15–0.14	−0.24–0.35
	QEs	6	3.10–105.01	2.95–14.95	−0.46–0.33	−0.1 to −0.02
Sum		1904	3.02–105.01	0.06–23.83	−0.46–0.41	−0.43–0.68

a Represents additive effect.

b Represents dominant effect

### Transcriptomic analysis

To identify the potential functional relevance of candidate genes, we integrated the transcriptome data with QTL-GWAS results. RNA-seq generated >6 Gb of high-quality data per sample (Q30 > 97%, GC > 44%). Clean reads showed high mapping rates (>78%) to the reference genome, ensuring reliable downstream analysis (Table S16). Genome-wide expression analysis identified 40 042 DEGs across four comparisons (Figs S9 and S10): BT vs BS (6348; 3229 up/3119 down), YSJ vs JHN (13 056; 6347 up/6709 down), YSJ vs BS (10 625; 4265 up/6360 down), and BT vs JHN (10 013; 5690 up/4323 down). Among these, 3208 genes were commonly regulated across all comparisons, while 596–1692 genes showed comparison-specific expression. GO enrichment analysis of DEGs revealed distinct functional patterns between drought-tolerant (YSJ/BT) and drought-sensitive (JHN/BS) genotypes (Fig. S11). The YSJ vs JHN comparison highlighted transmembrane transport, metabolic processes, and stress response pathways (Rich factor: 0.4–0.52, *P* < 0.01). The BT vs BS comparison enriched in photosynthesis, light harvesting, and membrane-related processes (Rich factor: 0.4–0.6, *P* < 0.01). Notably, membrane-associated processes and stress response mechanisms were commonly enriched across all comparisons, with UDP-glycosyltransferase activity and transmembrane transporter activity being particularly prominent (*P* < 0.001). These findings suggest that enhanced drought tolerance in YSJ and BT involves coordinated regulation of membrane integrity, stress signaling, and photosynthetic efficiency—key adaptive traits under water scarcity.

### Candidate genes for drought tolerance

Integration of QTL-GWAS loci identified 25 candidate genes within 100-kb flanking regions of significant SNPs (Table 9), including 18 near QTL-GWAS colocalized SNPs and seven adjacent to robust loci (Fig. S12; Table S17). Three genes (*Cn1062070*, *Cn0526230*, *Cn0377540*) were mapped to reference genome intervals, with *Cn1062070* located within major QTL qDS-3-82. Among the 25 candidates, 10 had annotated functions primarily in stress responses, including the drought-related NAC TF family member *CUC2* (corresponding to SNP S2_56531306) previously reported in chrysanthemum [51]. The *Arabidopsis LPAT5* homolog *Cn0631900* was mapped to MFVD-associated loci S5_240 361 573 and S5_240361644, functioning in lipid synthesis and salt tolerance through interaction with *BnWIN1* in *Brassica napus* [52]. At another MFVD locus, S8_25256427^*^, *Cn1297430* (*Arabidopsis ATL6* homolog) was identified, which cooperates with *ATL31* to enhance salt stress tolerance [53]. Phytohormone signaling networks were highlighted by *Cn0632390* (*CYP82C4* homolog) at WI loci S2_63233913/S2_63481521 involved in the jasmonic acid pathway, and *Cn1012440* (*DEL1* homolog) at PH locus S6_303583784 regulating salicylic acid-mediated immunity [54]. Multiple stress-responsive genes were detected across different trait-associated regions: *Cn1230400* (*HSP17.6C* homolog) at RL locus S9_30890821 involved in heat stress response [55]; *Cn0377540* (*TPS21* homolog) within the major QTL qDS-5-19 at SFW locus S5_28143190, mediating stress tolerance through ABA and ethylene signaling cascades [56]; and *Cn1411320* (*PRX52* homolog) at RDW locus S1_24438560 conferring aphid resistance [57]. Additionally, two salt stress regulators were identified at FRSR loci: *Cn0238240* (*CAC2* homolog) at S1_57209975 participating in fatty acid biosynthesis and oxidative stress response [58], and *Cn0437420* (*MAF4* homolog) at S1_247130487 functioning as a negative regulator of salt stress response [59].

**Table 6:** QTL hotspot distribution for drought tolerance identified by linkage analysis and GWAS across nine chromosomes

Hotspots	Chr	Interval (bp)	QTL/QTN number	Hotspots	Chr	Interval (bp)	QTL/QTN number
H1_25	1	240 593 700–249 843 316	11	H5_17	5	160 094 597–164 366 371	12
H2_21	2	200 518 728–207 285 804	10	H5_20	5	193 562 746–199 985 452	11
H2_27	2	262 785 823–268 462 180	10	H5_21	5	200 622 086–208 515 749	11
H2_33	2	320 921 203–328 388 773	12	H5_22	5	210 379 425–219 164 034	13
H2_41	2	402 308 949–409 614 054	11	H5_25	5	240 361 573–249 474 872	13
H3_12	3	103 601 468–109 912 518	10	H6_16	6	150 257 590–158 659 608	11
H3_19	3	181 211 170–189 810 260	11	H6_23	6	222 982 613–229 119 904	10
H3_20	3	191 269 177–199 911 931	16	H6_37	6	361 766 576–375 306 620	11
H3_28	3	270 469 777–279 371 201	11	H7_12	7	111 783 066–119 994 641	10
H3_30	3	290 596 713–299 833 018	10	H7_20	7	190 676 310–199 529 717	10
H4_1	4	955 325–8 448 187	10	H8_5	8	40 906 764–48 741 032	10
H4_29	4	281 366 182–289 794 021	10	H8_9	8	81 292 153–87 962 222	13
H5_4	5	30 285 974–39 026 603	12	H8_10	8	91 568 127–98 381 810	10
H5_6	5	50 332 046–59 807 236	11				

**Table 7:** The 31 colocalized QTL-GWAS loci for the drought tolerance-related traits

Trait	Sig. QTL	Env	Chr	Colocalized SNPs	Env
MFVD	*qDS-5-28*	BLUE	5	S5_40875826	BLUE
	*qDS-5-164*	E3	5	S5_240 361 573	E1
	*qDS-5-164*	E3	5	S5_240361644	BLUE
WI	*qDS-2-30*	BLUE	2	S2_56531306	Multi_QTN
	*qDS-2-34*	E3	2	S2_63233913	E1
	*qDS-2-34*	E3	2	S2_63481521	E3
	*qDS-5-190*	E1	5	S5_276803132	E2, E3, Multi_QTN
	*qDS-7-99*	E3	7	S7_198622580	E3
PH	*qDS-6-186*	E3	6	S6_303583784	E2
	*qDS-6-186*	E3	6	S6_305966617	E2, Multi_QTN
	*qDS-9-16*	BLUE	9	S9_19141466	Multi_QTN
RL	*qDS-2-147*	E1	2	S2_275021621	E1
	*qDS-8-222*	BLUE	8	S8_188962338	Multi_QTN
SFW	*qDS-2-28*	BLUE	2	S2_56427398	E2
	*qDS-5-19*	E1	5	S5_28143190	E1
	*qDS-5-19*	E1	5	S5_30446876	E1
	*qDS-6-137*	E3	6	S6_223677731	E1
RFW	*qDS-3-82*	E2,E3	3	S3_109 912 518	E3
	*qDS-3-82*	E2,E3	3	S3_111718348	E1
	*qDS-5-10*	E2	5	S5_17557470	E2
	*qDS-5-19*	E3	5	S5_22774330	E1
	*qDS-6-169*	E2	6	S6_204743538	E1, Multi_QTN
	*qDS-6-190*	E3	6	S6_315684905	Multi_QTN
SDW	*qDS-3-73*	E3	3	S3_97558905	BLUE
	*qDS-5-110*	E2	5	S5_161289584	E2
	*qDS-5-110*	E2	5	S5_163453497	BLUE, Multi_QTN
	*qDS-7-58*	BLUE	7	S7_118358128	BLUE, Multi_QTN
RDW	*qDS-5-11*	E3	5	S5_18233792	Multi_QTN
	*qDS-6-21*	E3	6	S6_33714276	E1
	*qDS-6-21*	E3	6	S6_37834731	BLUE
DRSR	*qDS-5-1*	E2	5	S5_8189150	BLUE

**Table 8:** The seven significant loci screened based on three other strict colocalization criteria

Marker	Trait	LOD (Q)	add^a^	dom^b^	R^2^ (%)	*P*-value	Sig.
S1_24438560	RDW3	32.11	−0.02	0.37	17.69	7.75E-33	SIG
	RDW.BLUE	13.94	0.01	0.16	3.89	1.15E-14	SIG
S8_25256427	MFVD2	16.40	−0.00	0.09	10.24	4.02E-17	SIG
	MFVD.BLUE	18.76	−0.00	0.09	13.71	1.73E-19	SIG
S1_247130487	FRSR1	36.18	−0.01	0.68	23.83	6.60E-37	SIG
	FRSR	18.01	−0.04	0.36	9.66	9.89E-19	SIG
S1_57209975	FRSR2	13.51	0.12	−0.02	8.86	3.13E-14	SIG
	FRSR3	18.75	0.13	−0.05	13.41	1.77E-19	SIG
	FRSR.BLUE	8.511	0.07	−0.06	4.04	3.09E-09	SIG
S4_10377630	DRSR3	22.05	0.14	0.00	11.55	8.92E-23	SIG
	DRSR	19.29	0.10	−0.14	7.45	5.16E-20	SIG
S5_6781238	RFW3	23.84	0.10	0.00	13.75	1.45E-24	SIG
	RFW.BLUE	12.43	0.06	−0.01	5.77	3.76E-13	SIG
	RFW	9.44	0.05	−0.01	2.74	3.65E-10	SIG
S9_30890821	RL3	7.24	−0.06	−0.01	7.36	5.81E-08	SUG
	RL.BLUE	14.36	−0.09	0.01	12.36	4.34E-15	SIG
	RL	6.93	−0.05	−0.02	2.13	1.18E-07	SUG

a Represents additive effect,

b Represents dominance effect

**Table 9:** Twenty-five candidate genes identified near 38 significant loci for drought tolerance

Trait	QTN	Env	Gene ID	Homologous gene in *Arabidopsis*			
				Gene	Symbol	Description	Annotation
MFVD	S5_40875826	BLUE					
	S5_240 361 573	E1	*Cn0631920*	*AT2G23780.3*		RING/U-box superfamily protein	
			*Cn0631900*	*AT3G18850.4*	*LPAT5*	lysophosphatidyl acyltransferase 5	It binds to *BnWIN1* in *B. napus* and participates in lipid synthesis and salt tolerance [52]
			*Cn0631890*	*AT5G42620.2*		metalloendopeptidase / zinc ion binding protein	
	S5_240361644	BLUE	*Cn0631920*	*AT2G23780.3*		RING/U-box superfamily protein	
			*Cn0631900*	*AT3G18850.4*	*LPAT5*	lysophosphatidyl acyltransferase 5	
			*Cn0631890*	*AT5G42620.2*		metalloendopeptidase / zinc ion binding protein	
	S8_25256427^*^	E2, BLUE	*Cn1297430*	*AT3G05200.1*	*ATL6*	RING/U-box superfamily protein	*ATL6* and *ATL31* positively modulate *Arabidopsis* tolerance to salt stress [53]
WI	S2_56531306	Multi_QTN	*Cn0026670*	*AT5G53950.1*	*CUC2*	NAC domain transcriptional regulator superfamily protein	It is related to the drought tolerance of chrysanthemum [51]
	S2_63233913	E1	*Cn0632390*	*AT4G31940.1*	*CYP82C4*	cytochrome P450%2C family 82%2C subfamily C%2C polypeptide 4	In response to abiotic stresses in plants and involved in the metabolism or signal transduction of plant hormones, especially pathways related to jasmonic acid [54]
	S2_63481521	E3	*Cn0632390*	*AT4G31940.1*	*CYP82C4*	cytochrome P450%2C family 82%2C subfamily C%2C polypeptide 4	
	S5_276803132	E2, E3, Multi_QTN	*Cn0907630*	*AT5G50090.1*		D-ribose-binding periplasmic protein	
			*Cn0907620*	*AT5G62890.3*		Xanthine/uracil permease family protein	
	S7_198622580	E3	*Cn0002440*	*AT1G28110.2*	*SCPL45*	serine carboxypeptidase-like 45	Not exactly reported
PH	S6_303583784	E2	*Cn1012440*	*AT3G48160.2*	*DEL1*	DP-E2F-like 1	
	S6_305966617	E2, Multi_QTN	*Cn1013190*	*AT3G29670.1*	*PMAT2*	HXXXD-type acyl-transferase family protein	Not exactly reported
	S9_19141466	Multi_QTN	*Cn0268150*	*no hit*			
RL	S2_275021621	E1					
	S8_188962338	Multi_QTN	*Cn1307350*	*AT4G24060.1*	*DOF4.6*	Dof-type zinc finger DNA-binding family protein	Not exactly reported
	S9_30890821^*^	E3,BLUE, Multi_QTN	*Cn1230400*	*AT1G53540.1*	*HSP17.6C*	HSP20-like chaperones superfamily protein	Involved in high temperature stress of *Arabidopsis thaliana* [55]
SFW	S2_56427398	E2	*Cn0162090*	*no hit*			
	S5_28143190	E1	*Cn0377540*	*AT5G23960.2*	*TPS21*	HSP20-like chaperones superfamily protein	Regulates stress resistance through hormone signaling of ABA and ethylene [56]
	S5_30446876	E1	*Cn0376530*	*no hit*			
	S6_223677731	E1					

(Continued)

**Table 9:** Continued

Trait	QTN	Env	Gene ID	Homologous gene in *Arabidopsis*			
				Gene	Symbol	Description	Annotation
RFW	S3_109 912 518	E3	*Cn1062070*	*AT4G16210.1*	*ECHIA*	enoyl-CoA hydratase/isomerase A	Not exactly reported
	S3_111718348	E1	*Cn1062070*	*AT4G16210.1*	*ECHIA*	enoyl-CoA hydratase/isomerase A	Not exactly reported
	S5_17557470	E2					
	S5_22774330	E1					
	S6_204743538	E1, Multi_QTN					
	S5_6781238*	E3,BLUE, Multi_QTN	*Cn0093410*	*AT5G55670.1*		RNA-binding (RM/RBD/RNP motifs) family protein	
SDW	S6_315684905	Multi_QTN					
	S3_97558905	BLUE					
	S5_161289584	E2					
	S5_163453497	BLUE, Multi_QTN					
	S7_118358128	BLUE, Multi_QTN					
RDW	S5_18233792	Multi_QTN	*Cn0526230*	*no hit*			
	S6_33714276	E1					
	S6_37834731	BLUE	*Cn1101880*	*AT3G03960.1*	*CCT8*	TCP-1/cpn60 chaperonin family protein	Not exactly reported
	S1_24438560*	E3, BLUE	*Cn1411320*	*AT5G05340.1*	*PRX52*	Peroxidase superfamily protein	Involvement in soybean aphid resistance [57].
FRSR	S1_57209975*	E2, E3, BLUE	*Cn0238240*	*AT5G35360.1*	*CAC2*	acetyl Co-enzyme a carboxylase biotin carboxylase subunit	Response to salt stress and oxidative stress [58].
	S1_247130487*	E1, Multi_QTN	*Cn0437420*	*AT5G65070.5*	*MAF4*	K-box region and MADS-box transcription factor family protein	Involved in negative regulation of salt stress [59].
DRSR	S4_10377630*	E3, Multi_QTN	*Cn1152600*	*AT2G35040.1*		AICARFT/IMPCHase bienzyme family protein	
	S5_8189150	BLUE					

**Figure 5:**
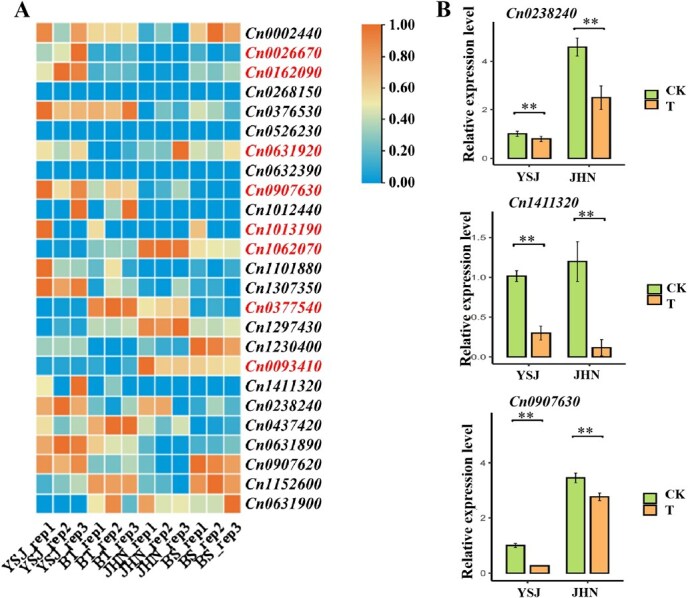
Identification and qRT-PCR validation of drought-responsive candidate genes. (A) Heatmap showing the expression profiles of 25 candidate genes associated with drought tolerance. (B) Relative expression levels of three selected candidate genes in drought-tolerant parents YSJ and JHN under control (CK) and treatment (T) conditions as determined by qRT-PCR (*P* < 0.01). The eight high-priority candidate genes from initial screening are marked in red.

Based on the RNA-seq results, we identified eight DEGs from the 25 candidate genes (FPKM ≥ 3, differential expression in ≥2 comparisons; Fig. 5A; Table S17). These DEGs exhibited distinct expression patterns between drought-tolerant (YSJ, BT) and drought-sensitive (JHN, BS) genotypes. *Cn1062070* showed the most dramatic changes with significant downregulation in tolerant genotypes (log_2_FC = −2.41 in YSJ vs JHN). Similarly, *Cn0093410* and *Cn0631920* were downregulated in tolerant genotypes. In contrast, *Cn0026670* and *Cn0162090* displayed marked upregulation in YSJ (log_2_FC > 2.9), while Cn0907630 maintained high expression in both tolerant lines (FPKM >35). Notably, *Cn0377540* showed genotype-specific expression with strong upregulation in BT (log_2_FC = 3.69) but downregulation in YSJ, suggesting divergent regulatory mechanisms. qRT-PCR validation of three randomly selected candidate genes confirmed high consistency with RNA-seq data (*P* < 0.01, Fig. 5B), reinforcing the reliability of transcriptome profiling.

### Haplotype and expression profiling of *Cn1062070*

**Figure 6:**
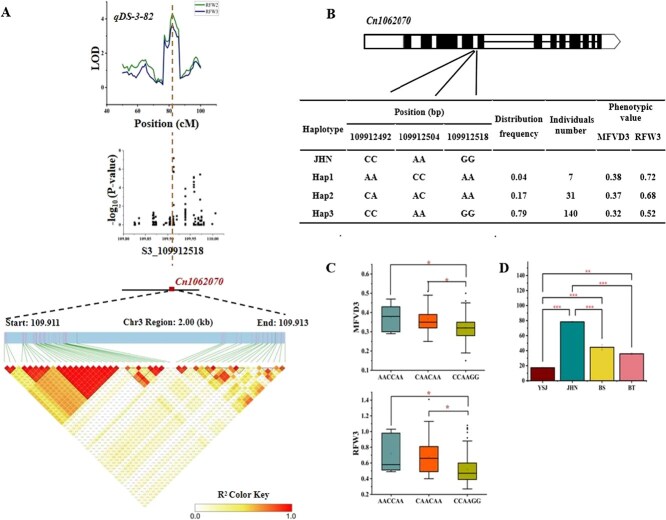
Haplotype analysis of drought-responsive candidate gene *Cn1062070*. (A) Genomic localization and LD block of *Cn1062070*. (B) Structure and haplotypes of *Cn1062070*. (C) Phenotypic differences in corresponding MFVD3 and RFW3 among the three haplotypes. (D) Differential expression patterns of *Cn1062070* in four pools (JHN, YSJ, BS, and BT) under control (CK) and drought treatment (T) conditions. ^*^ and ^**^ indicate significant expression changes at *P* < 0.05, and *P* < 0.01, respectively.

The candidate gene *Cn1062070* harbored three polymorphic sites, Chr3_109912492, Chr3_109912504, and Chr3_109 912 518, in its intronic region (Fig. 6A and B). The locus Chr3_109 912 518, located within the major QTL qDS-3-82 for RFW under E2 and E3 conditions, showed significant associations with MFVD3 and RFW3 in GWAS. Haplotype analysis identified three distinct haplotypes: Hap1 and Hap2 occurred at low frequencies of 0.04 and 0.17, respectively, while Hap3 showed the highest frequency (0.79), identical to that of the *C. nankingense* reference genome (Fig. 6B). Phenotypic analysis revealed that the rare Hap1 and Hap2 exhibited higher MFVD3 and RFW3, whereas the dominant Hap3 exhibited the lowest MFVD3 (0.32) and RFW3 (0.52). Based on the *Arabidopsis* genomic database, *Cn1062070* is a homolog of *ECHIA* (enoyl-CoA hydratase/isomerase A), but the function remains uncharacterized. Noteworthily, the *Cn1062070* exhibited divergent expression profiles between parental lines and marked induction under drought stress, probably indicating its pivotal role in regulating drought tolerance.

## Discussion

Drought is a major abiotic stress factor affecting plant growth, development, and yield. The increasing scarcity of freshwater resources poses a significant challenge to the sustainable development of agriculture. In chrysanthemums, water deficit conditions hinder plant growth and development, decreasing ornamental quality and yield [60–62]. Breeding varieties with enhanced drought tolerance is a significant strategy for dealing with drought adversity. However, more knowledge of the inheritance of drought tolerance is needed to ensure the improvement of drought tolerance. In this study, we integrated linkage mapping, GWAS, and RNA-seq to explore the genetic architecture and identify essential loci of drought tolerance in reciprocal hybrid progeny from *C. dichrum* (drought-tolerant, YSJ) and *C. nankingense* (drought-sensitive, JHN). The results add a new understanding of the genetic mechanisms underlying drought tolerance in chrysanthemum species and offer critical genetic resources for future cultivar improvement.

Multiple studies have confirmed that drought tolerance is a complex quantitative trait controlled by multiple genes [6, 10, 13]. In this study, phenotypic variation in drought tolerance-related traits was markedly higher in the reverse cross JHN × YSJ compared to the forward cross YSJ × JHN, and reciprocal effects were observed across all traits, except for WI, PH, SFW, and SDW, with root-related traits showing the most pronounced divergence. These patterns implicate the crucial role of cytoplasmic inheritance and maternal effects. The directional differences in trait expression, exemplified by the superior performance of the JHN × YSJ progeny, further suggest genomic imprinting as a regulatory mechanism. Despite bidirectional transgressive segregation, reciprocal hybrids predominantly displayed negative heterosis for most drought tolerance-related traits. As elucidated in our recent research [59], this heterosis decline can be attributable to the intrinsic heterozygosity of parental genomes.

Recent advances in chrysanthemum genetic mapping have provided robust frameworks for dissecting the genetic architecture of resistance and ornamental traits [60–62]. Here, we constructed the first high-density genetic map from an interspecific *Chrysanthemum indicum* × *C. morifolium* F_1_ population, comprising 6677 high-quality SNPs and an average density of 0.28 cM. The ICIM analysis identified 89 drought-responsive QTLs, including 14 pleiotropic loci affecting multiple traits. Lately, the 3VmrMLM software has emerged as a powerful tool demonstrating robust performance in the detection of stable small-effect loci across environments and the dominance and genotype–environment interactions [12, 63, 64]. Leveraging the 3VmrMLM, we identified 1360 significant QTNs in Single_Env analysis, and 394 QTNs, 114 QEIs, and 6 QEs in Multi_Env analysis for drought tolerance-related traits. The analysis of the genetic effects of QTLs and QTNs provides insights into the decline of hybrid vigor. Over 90% of the detected loci showed a low PVE < 10%, with dominance effects predominating in key traits, namely WI, PH, SFW, SDW, FRSR, and DRSR. Some of the epistatic QEs for WI exhibited antagonistic or synergistic effects, while most loci showed unfavorable genetic effects, mirroring the reduced heterosis observed in reciprocal progeny. These findings reveal the genetic complexity of drought tolerance in chrysanthemum species, shaped by polygenic interactions and context-dependent allele effects.

The integration of QTL mapping and GWAS provides a practical approach to candidate gene identification [13, 65]. Through QTL-GWAS colocalization, we identified 38 significant SNPs within drought-responsive genomic regions and three stringent filtering criteria in GWAS, leading to the annotation of 10 characterized and 15 novel candidate genes (Table 9) based on diploid *C. nankingense* genome and *Arabidopsis* genomic database. Based on the bulked RNA-seq analysis, we found eight out of the 25 candidate genes showed contrasting expression patterns, and some were preliminarily verified by qRT-PCR. The outcome provides valuable genetic resources for elucidating molecular mechanisms regulating drought tolerance in chrysanthemum species.

Haplotype analysis enables the identification of trait-associated variants for marker-assisted breeding. In chrysanthemum, Lou *et al*. [64] revealed two elite *CmARF16* haplotypes with a smaller leaf angle, and Su *et al*. [66] identified a superior *CmERF-1* haplotype showing late flowering time. The current work detected three haplotypes in the intronic region of the candidate gene *Cn1062070*, an *Arabidopsis ECHIA* homolog. Compared to the predominant Hap3, the rare Hap1 and Hap2 showed higher values for drought tolerance traits. Thus, we considered the Hap1 and Hap2 superior haplotypes despite further functional validation being necessary to examine whether the haplotypes are suitable to serve as molecular markers for drought tolerance breeding*.* Emerging evidence highlights the functional roles of intronic variants in gene regulation, complementing well-documented coding region polymorphisms. For instance, a single intronic variation can regulate the flowering time of *Arabidopsis* via *COOLAIR* splicing [67] or through modulating both *FLM* expression and intron splicing [68]. Haplotype analysis revealed that intronic insertions in *HvHKT1;5* enhanced root expression and ion homeostasis, conferring improved salt tolerance in barley [69]. Proteogenomic analysis of sweet potato validated many intronic *ORFs* as novel protein-coding genes and identified considerable loci for annotation refinement, demonstrating the functional significance of intronic regions [70]. However, further evidence is needed to elucidate the haplotype associations of the intronic SNP within *Cn1062070* and its putative TF binding interactions.

## Materials and methods

### Mapping population

A total of 273 F_1_ hybrids, derived from reciprocal crosses between drought-tolerant *C. dichrum* (YSJ) and drought-sensitive *C. nankingense* (JHN), were utilized for genetic map construction. The reciprocal F_1_ population consisted of 177 hybrid individuals from the forward cross YSJ × JHN and 96 from the reverse cross JHN × YSJ. All materials were planted in the Chrysanthemum Germplasm Resource Preservation Center at Nanjing Agricultural University, China.

### Estimation of drought tolerance

We conducted a 3-year evaluation of drought tolerance for the mapping population in greenhouse conditions during 2020–22 (E1, E2, and E3, respectively). The experiment employed a completely randomized block design comprising a control group (CK; soil moisture maintained at ~75%) with two replicates and a drought-treated group (T) with three replicates. Each replicate included six plants. Following a 14-day drought treatment period, drought tolerance-related traits including wilting index (WI), plant height (PH, cm), root length (RL, mm), shoot fresh weight (SFW, g), root fresh weight (RFW), shoot drought weight (SDW, g), root drought weight (RDW, g), fresh weight root shoot ratio (FRSR), dry weight root shoot ratio (DRSR) were assessed for each genotype. PH was measured along the shoot from base to apex after cutting the shoots at the soil surface. RL was determined by measuring the length from the root–shoot junction to the tip of the longest root. Fresh and dry weights of cleaned roots and shoots were used to calculate FRSR (RFW/SFW) and DRSR (RDW/SDW). Plant drought tolerance was evaluated using drought coefficient (DC = T/CK), eliminating baseline genotypic differences. The membership function method [29] was employed to determine each trait's membership function value of drought tolerance (MFVD). The average MFVD represents the drought tolerance for each genotype.

Statistical analyses were performed using IBM SPSS Statistics 25 (IBM Corp., 2017). Broad-sense heritability was calculated as H^2^ = *VG/VP*, where *VG* and *VP* represent genetic and phenotypic variance, respectively. Mid-parent heterosis (MPH) was estimated as MPH (%) = [(F_1_ – MP)/MP] × 100, where F_1_ and MP denote F_1_ hybrid performance and mid-parent value, respectively. Reciprocal effects were calculated as RE = (JHN × YSJ) – (YSJ × JHN). Violin plots and correlation heatmaps were generated using Origin 2021 (OriginLab Corp., USA) and R 4.0.2, respectively. Multiple comparisons were conducted using Scheffe's test at *P* < 0.05.

### DNA extraction and genotyping

Genomic DNA was extracted from parental lines and the 273 reciprocal F_1_ progenies using a modified cetyltrimethylammonium bromide (CTAB) method [30]. DNA concentration and purity were determined by a NanoDrop 2000 UV–vis spectrophotometer and checked on 1% agarose gels. GBS was conducted following the method described by Elshire *et al*. [31], achieving an average sequencing depth of 6X. Clean sequencing reads were aligned to the diploid *C. nankingense* reference genome [26] using Burrows–Wheeler Aligner (BWA-MEM v0.7.10) [32]. SNP calling was performed using Genome Analysis Toolkit (GATK) pipelines according to standard procedures recommended by GATK best practices [33]. The identified variants were subsequently filtered to remove SNPs with minor allele frequency (MAF) <0.05 and missing data rate >10%.

### Genetic linkage map construction and QTL mapping

The resulting high-quality SNP markers were categorized into eight segregation patterns: ‘ab × cd’, ‘nn × np’, ‘hk × hk’, ‘ef × eg’, ‘cc × ab’, ‘aa × bb’, ‘ab × cc’, and ‘lm × ll’ [34, 35]. Considering the characteristics of the cross-pollination (CP) population, markers falling into three categories, ‘nn × np’, ‘hk × hk’, and ‘lm × ll’, were chosen for genetic linkage map construction. Furthermore, molecular markers containing abnormal bases or significant segregation distortion (Chi-squared test, *P* < 0.001) were excluded from subsequent analysis. To eliminate redundancy and potential sequencing errors, SNPs were binned using a sliding window approach [36].

Genetic linkage map construction and QTL mapping were performed using the genetic analysis of clonal F_1_ and double cross populations (GACD) software [37]. The number and patterns of informative alleles in parental lines and the F_1_ progeny were encoded, and parental linkage maps, as well as a consensus linkage map, were generated using the consensus double-cross mapping (CDM) function. Markers were then grouped and ordered utilizing the nnTwoOpt algorithm, with further refinement of the ordered chromosome accomplished through the sum of adjacent recombinant frequencies (SARF), employing a sliding window of five markers.

QTL mapping was conducted using an inclusive composite interval mapping (ICIM) algorithm to locate additive (a) and dominant (d) genes. The logarithm of the odds (LOD) threshold was calculated by 1000 permutation tests, with a significance level of *P* < 0.05. The potential position of the QTL was described based on the LOD peak position and its surrounding region.


\begin{equation*} \textrm{a}= \{\textrm{mu}(a\textrm{c}) - \textrm{mu}(\textrm{bd})\}/2; \end{equation*}

$$ \textrm{d} = \{\textrm{mu}(a\textrm{d}) + \textrm{mu}(\textrm{bc})\}/2 - \{\textrm{mu}(a\textrm{c}) + \textrm{mu}(\textrm{bd})\}/2 $$

where ‘a’ and ‘d’ are the additive and dominance effects. The mu(ac) and mu(bd) are the phenotypic means for the heterozygous loci having alleles from the same species, and mu(ad) and mu(bc) are the phenotypic means for the heterozygous loci carrying alleles from both species [34]. In addition, quantitative trait loci (QTLs) that share the same genomic position and are linked to distinct traits are regarded as identical QTLs.

### Genome-wide association study

To further elucidate the genetic architecture of drought tolerance, we conducted GWAS on the reciprocal F_1_ population comprising 273 progenies. Population structure was analyzed using the ADMIXTURE software [38] based on the high-quality SNP dataset. For GWAS, drought tolerance-related traits were analyzed separately across three environments (E1, E2, E3) and their Best Linear Unbiased Estimation (BLUE) values to identify Quantitative Trait Nucleotides (QTNs) using the ‘Single_Env’ function in the 3VmrMLM software [39]. Subsequently, we employed the ‘Multi_env’ function in 3VmrMLM for joint analysis of QTN-by-environment interactions (QEIs) and QTN-by-QTN epistatic effects (QEs) underlying drought tolerance. The stringent significance threshold was set at *P* = .05/m (2.48 × 10^−8^), where m represents the number of SNPs, for identifying significant QTNs, QEIs, and QEs. To avoid missing essential loci, a supplementary threshold of LOD > 3.0 was implemented to determine the suggested QTNs or QEIs [40]. Visualization of association results was achieved using the CMplot package in R [41], generating Manhattan plots to assess significance patterns and model fit.

### Transcriptome profiling

To identify drought-responsive differentially expressed genes (DEGs) and prioritize candidate genes associated with significant QTLs/QTNs, we conducted transcriptome profiling on parental lines (YSJ and JHN) and two extreme bulked pools: drought-tolerant (BT) and drought-sensitive (BS). Each pool comprised eight interspecific F_1_ hybrids exhibiting contrasting drought tolerance phenotypes. Plants underwent simulated drought stress in culture dishes with three replicates per treatment. Parental lines and selected extreme hybrids were subjected to ~6-h drought treatment until visible drought stress symptoms developed. Total RNA was extracted from the third to fourth fully expanded leaves of parental lines and the extreme hybrid genotypes using the pBIOZOL reagent (BioFlux, BSC55M1), with three biological replicates per sample. RNA quality and quantity were assessed using a NanoDrop 8000 spectrophotometer, and RNA integrity was confirmed through electrophoresis on the Agilent 2100 Bioanalyzer. High-quality RNA samples (RNA integrity number, RIN ≥ 7.0) were processed for library preparation. RNA-seq libraries were constructed and subjected to 100-bp paired-end sequencing on the Illumina NovaSeq platform. Clean reads were obtained using fastp [42] by removing reads with >10% N bases or > 50% low-quality bases (Q ≤ 19), followed by alignment to *C. nankingense* reference genome using HISAT2 software [43]. Gene expression levels were quantified as fragments per kilobase of transcript per million mapped reads (FPKM). DEGs between groups were identified using DESeq2 v1.6.3 [44], with thresholds set as false discovery rate (FDR) ≤0.01 and |log_2_FC| ≥ 1. Subsequently, Gene Ontology (GO) enrichment analysis of DEGs was performed using TBtools v1.076 software [45], and significantly enriched GO terms (FDR ≤ 0.05, Benjamini–Hochberg method) associated with drought response were visualized for biological interpretation.

### Colocalized significant SNPs and candidate gene prediction

The integration of GWAS and QTL mapping revealed significant colocated SNPs within drought tolerance loci. To prevent the omission of essential loci, we also applied the following three criteria for the selection of significant SNP loci: (1) QTNs colocalized in ≥2 datasets (R^2^ > 10%) in Single_Env analysis; (2) QTNs identified by both Single_Env and Multi_Env analyses (R^2^ > 10%); (3) Loci detected as both QTNs and QEIs in Multi_Env analyses. Candidate genes were screened within ±100 kb flanking regions [46] of significant SNPs based on the *C. nankingense* reference genome. These genes were further filtered for drought-responsive expression with FPKM ≥ 3 and |log_2_FC| ≥ 1 in at least two comparisons from transcriptome profiling. Gene functions were annotated by BLASTN searches against the *Arabidopsis* genome database (TAIR, https://www.arabidopsis.org) using BEDtools [47], prioritizing orthologs with known biological relevance to stress responses.

### qRT-PCR validation of candidate genes

Candidate genes were verified via quantitative real-time polymerase chain reaction (qRT-PCR) using RNA isolated from drought-stressed parental lines (YSJ and JHN). Primer sequences were retrieved from the qPCR primer database (https://biodb.swu.edu.cn/qprimerdb/) and validated for specificity using NCBI Primer-BLAST (https://www.ncbi.nlm.nih.gov/tools/primer-blast/). Relative expression levels were analyzed using the 2^−ΔΔCT^ method [48], with *CmEF1α* as the internal control. All experiments were performed in biological triplicates, and primer sequences are summarized in Table S1.

### Haplotype analysis

Haplotype blocks of key candidate genes were estimated using PLINK v1.9 [49] and Haploview v4.2 [50]. Significant associations between haplotypes and drought tolerance traits were assessed via ANOVA, followed by the identification of superior haplotypes displaying higher drought tolerance.

## Supplementary Material

Web_Material_uhaf169

## Data Availability

All data generated or analyzed in this study are included in the main text and its supplementary information files.
