# Supplementary material for: Integrative linkage mapping, GWAS, and RNA-Seq analysis unravel the genetic architecture and candidate genes for drought tolerance in Chrysanthemum interspecific F1 progeny
Source: Hortic Res. 2025 Jun 25;12(10):uhaf169. doi: 10.1093/hr/uhaf169 (PMC12528654; doi:10.1093/hr/uhaf169)
Supplement: Web_Material_uhaf169 [file web_material_uhaf169.zip › Supplement Figure S1-S12.pdf]

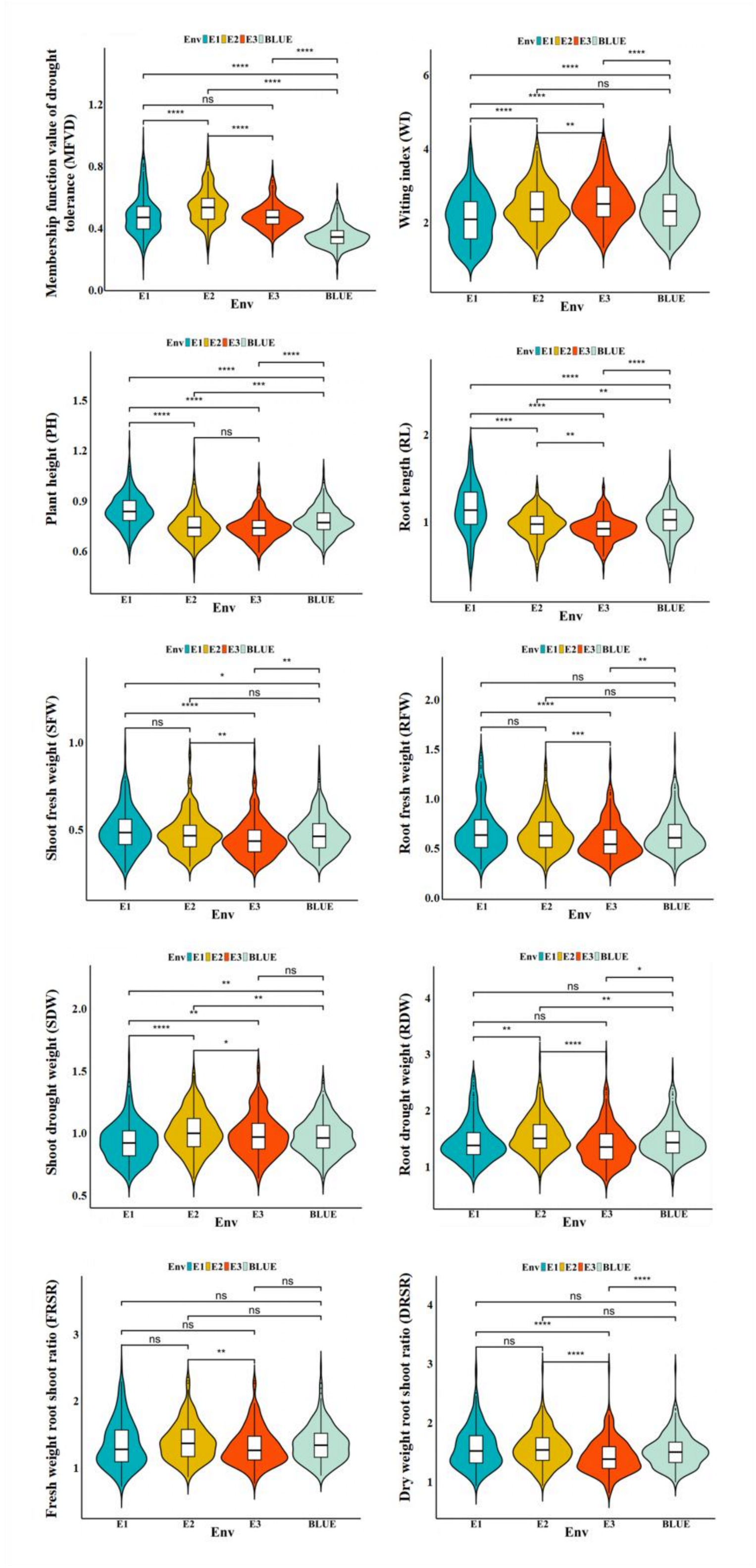

Figure S1 Distribution of 10 drought tolerance-related traits in reciprocal F_1 progenies of *C. dichrum* and *C. nankingense* under E1, E2, E3, and BLUE

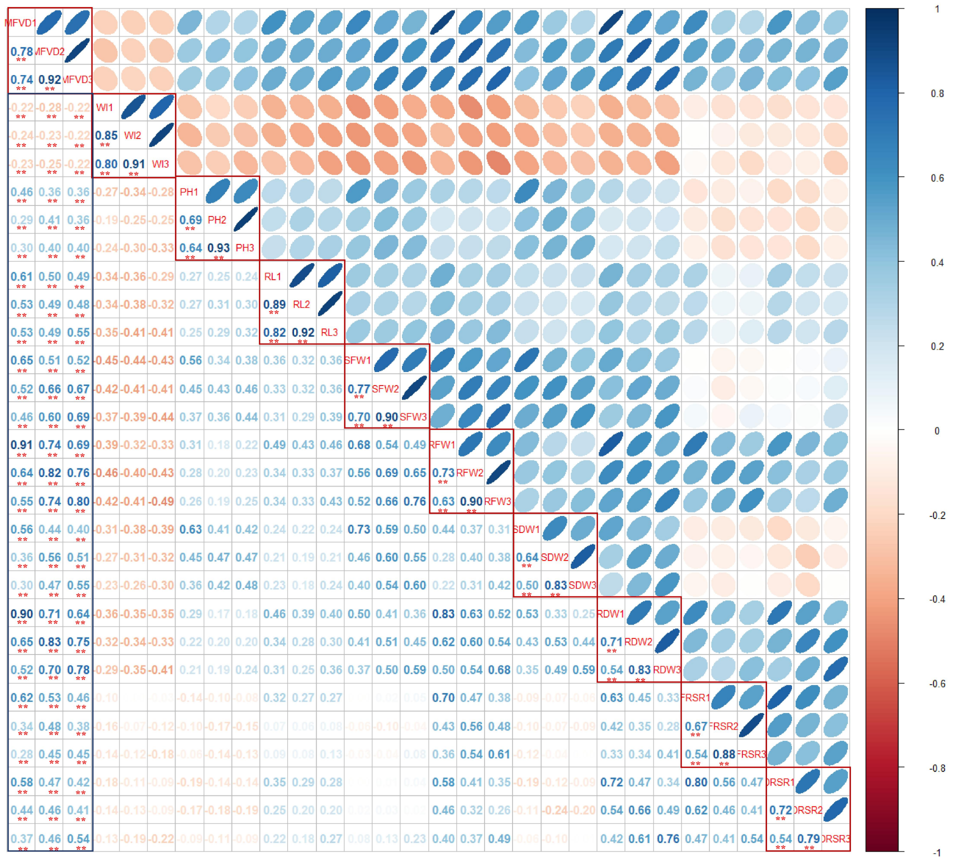

Figure S2 Correlation coefficients of BLUE values for drought tolerance-related traits among E1, E2, and E3 in the reciprocal F_1 hybrids of *C. dichromum* and *C.nankingense.* * and ** represent statistical significances at 0.05 and 0.01 levels, respectively.

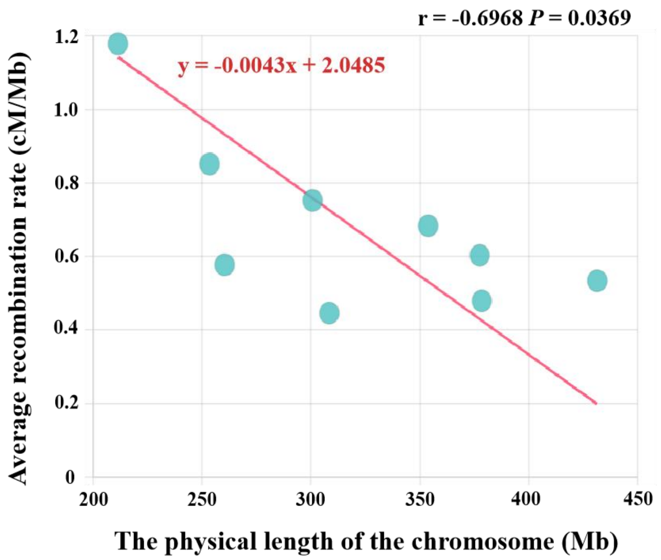

Figure S3 The relationship between recombination rate and physical length of the chromosomes.

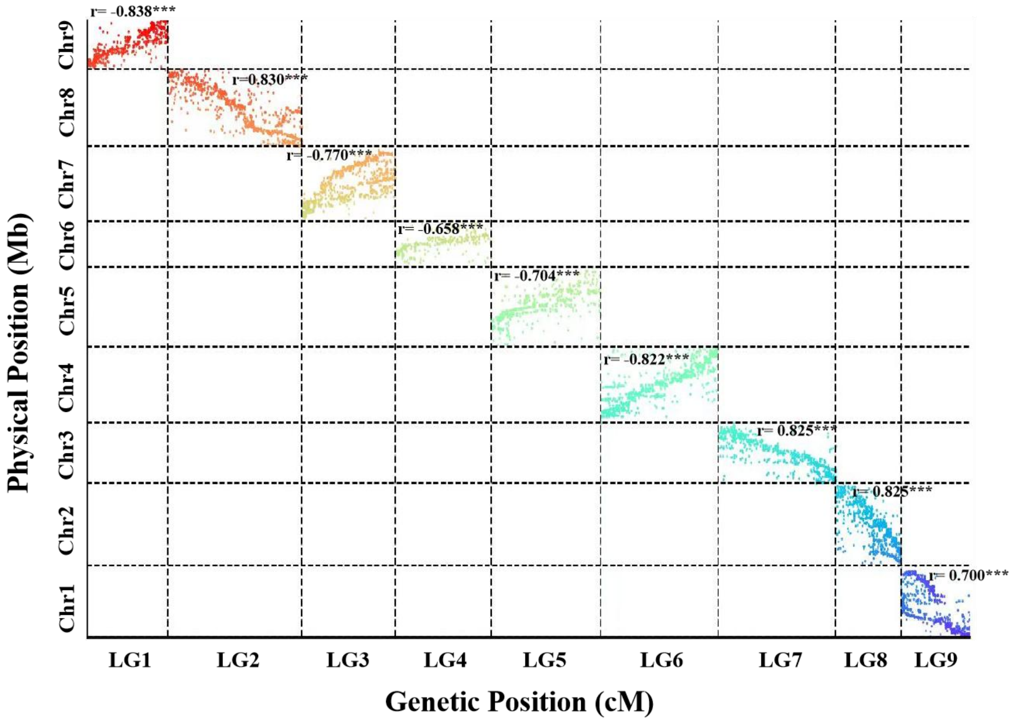

Figure S4 Collinearity scatter plot between the genetic map and the reference genome. The x-axis represents the genetic distance (cM) of each linkage group, and the y-axis indicates the physical position (Mb) in the *C. nankingense* reference genome. Different colors represent the nine linkage groups (LG1-LG9). Correlation coefficients (r) for each linkage group are indicated (***, $P < 0.001$).

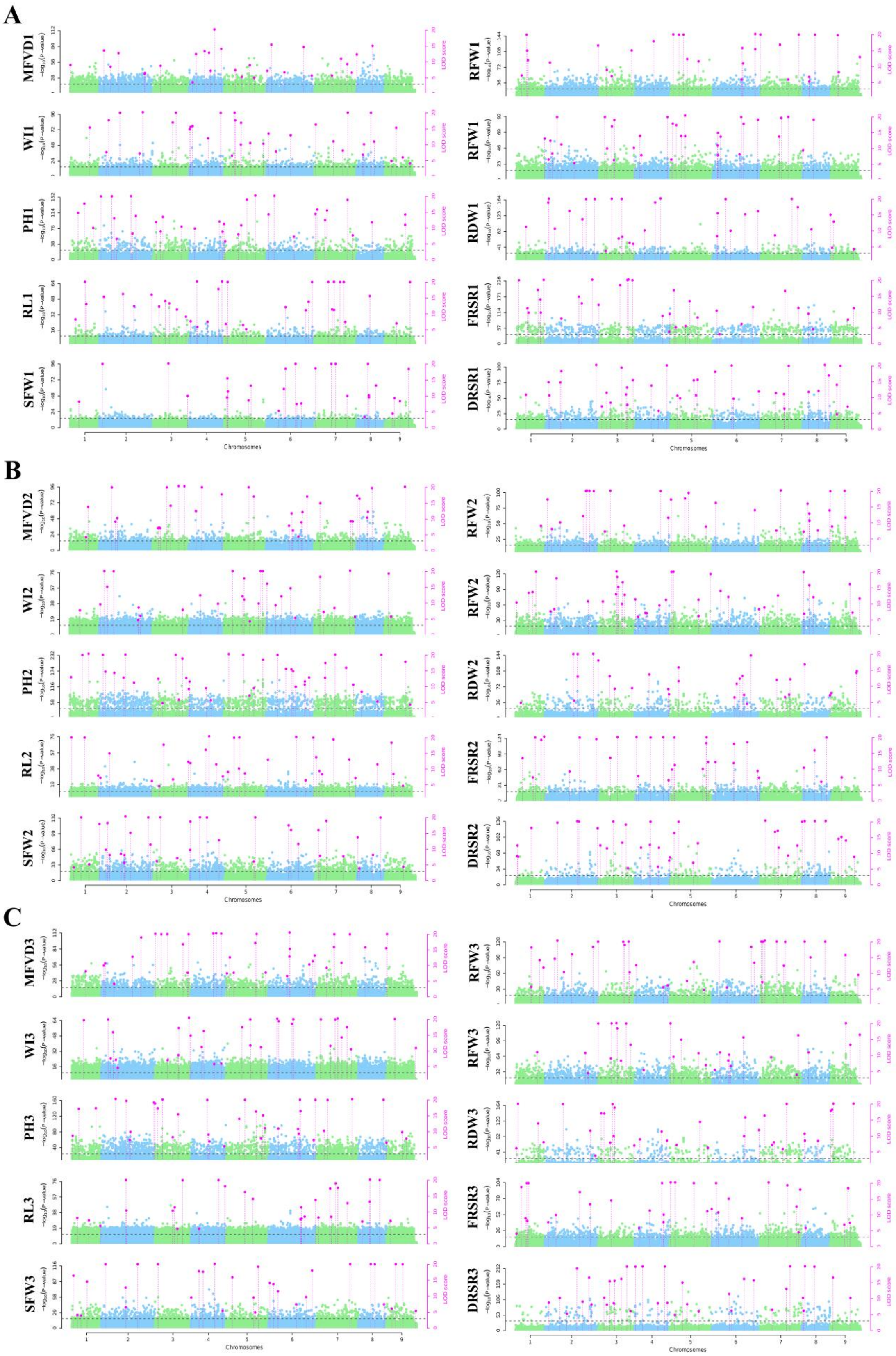

Figure S5 Manhattan plots of significant QTNs associated with 10 drought tolerance traits detected by Single_Env algorithm in 3VmrMLM software. A-C represent environments E1, E2 and E3, respectively. The horizontal lines indicate the LOD threshold of 3.0 for suggested loci. Pink dots

represent significant loci that passed both LOD ≥ 3.0 and Bonferroni correction thresholds.

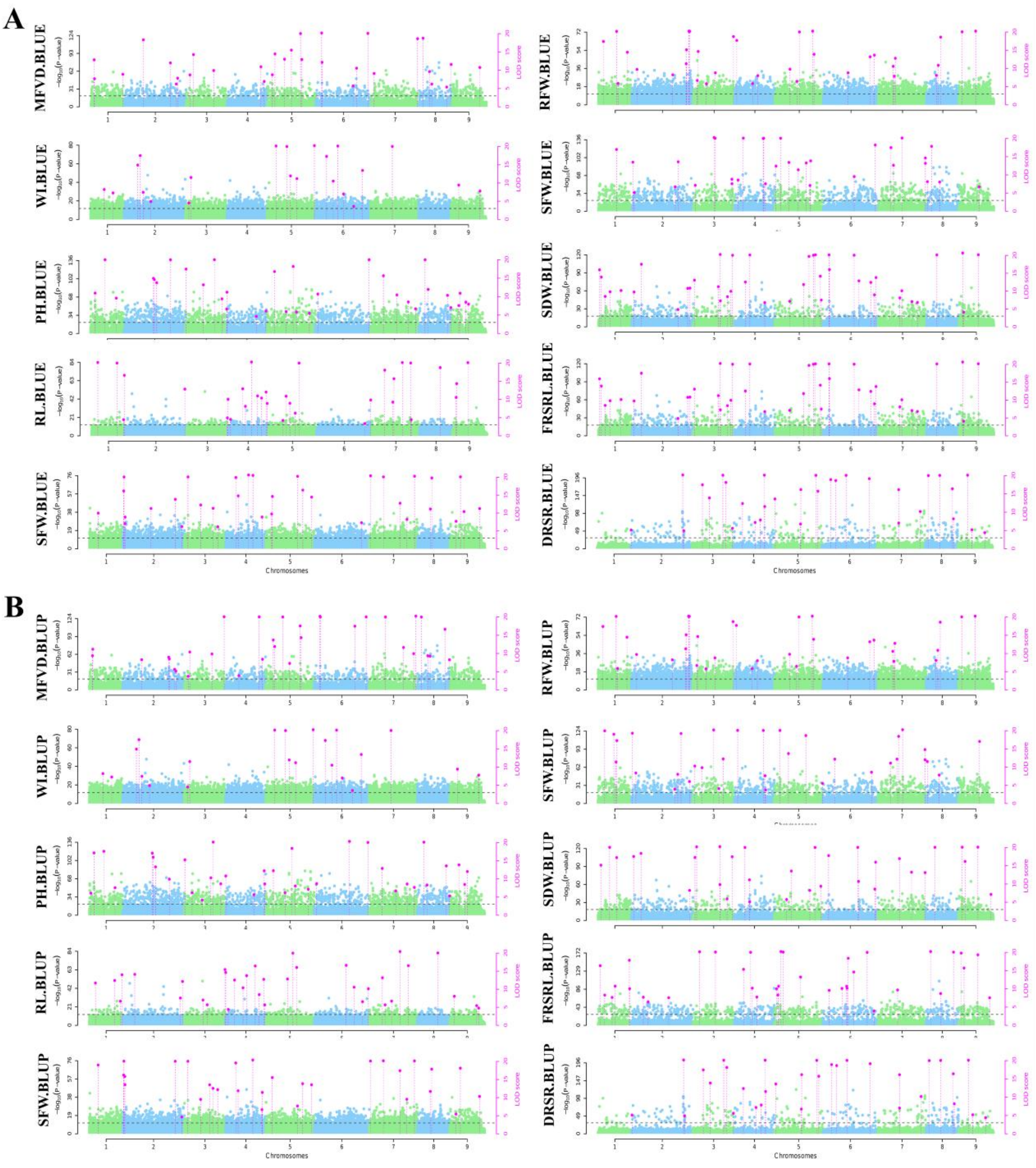

Figure S6 Manhattan plots of significant QTNs associated with 10 drought tolerance traits detected by Single_Env algorithm in 3VmrMLM software. A and B represent environments BLUE and BLUP, respectively. The horizontal lines indicate the LOD threshold of 3.0 for suggested loci. Pink dots represent significant loci that passed both LOD ≥ 3.0 and Bonferroni correction thresholds.

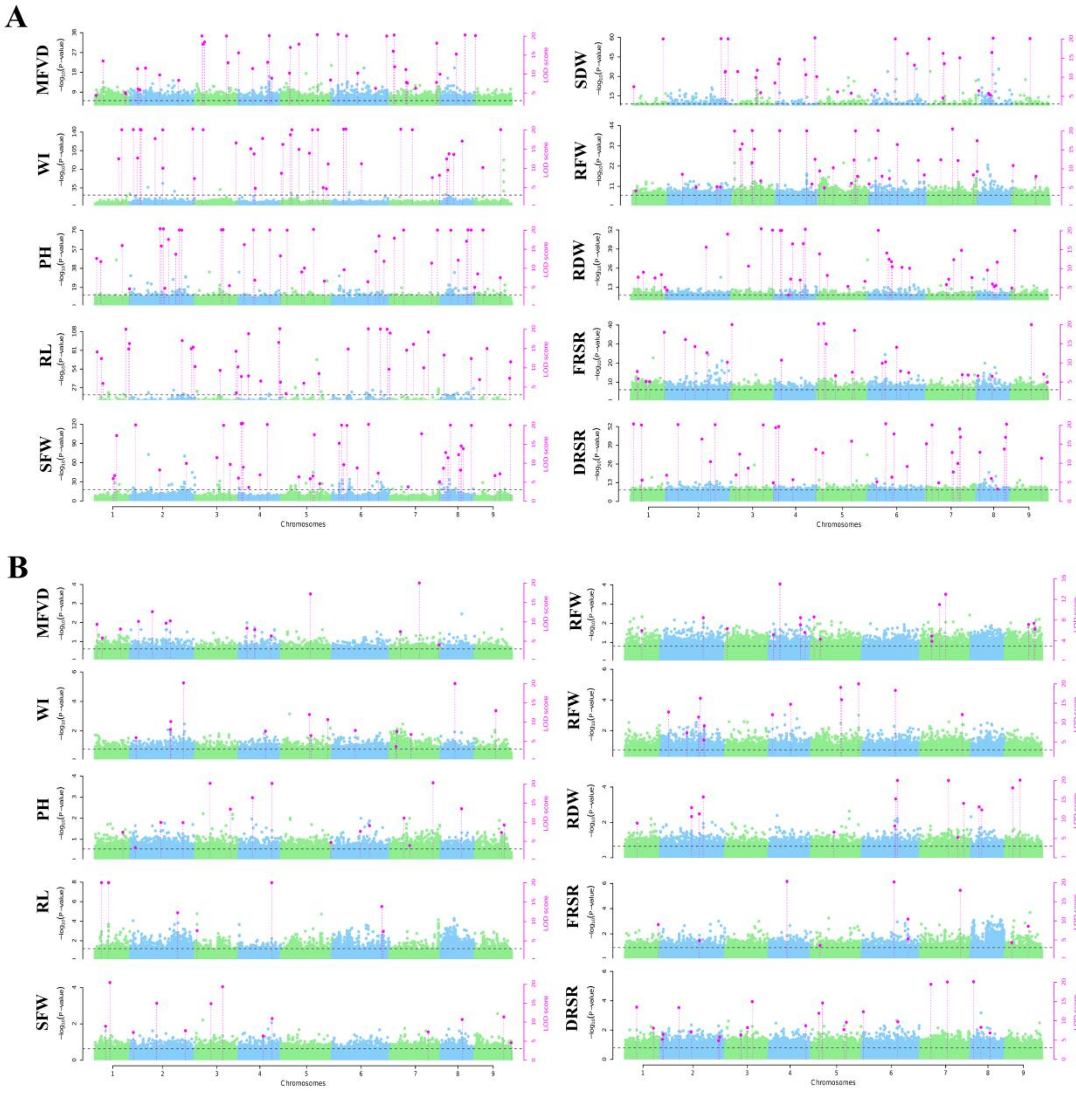

Figure S7 Manhattan plots of significant QTNs and QEIs associated with 10 drought tolerance traits detected by Multi_Env algorithm in 3VmrMLM software. A and B represent plots for significant QTNs and QEIs, respectively. The horizontal lines indicate the LOD threshold of 3.0 for suggested loci. Pink dots represent significant loci that passed both LOD ≥ 3.0 and Bonferroni correction thresholds.

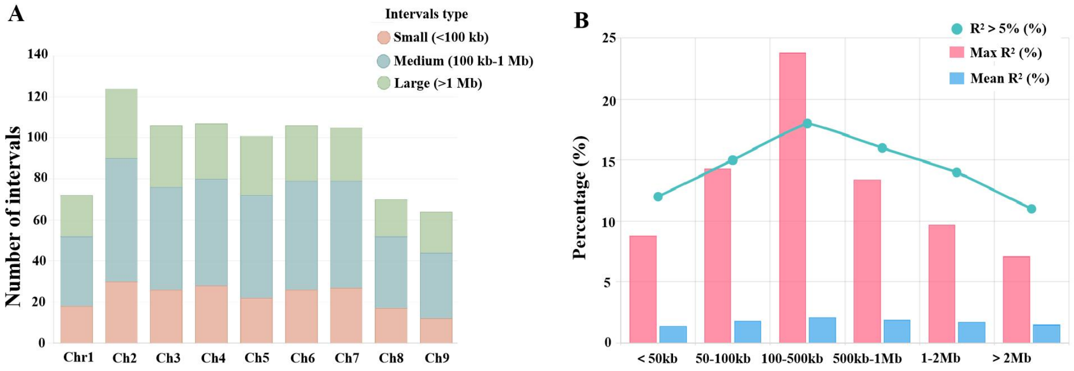

Figure S8 Size distribution of significant QTN Intervals identified by GWAS using 3VmrMLM software. (A) Size distribution of significant QTNs across 9 chromosomes. (B) Relationship between QTN interval size and phenotypic variation explained (R^2).

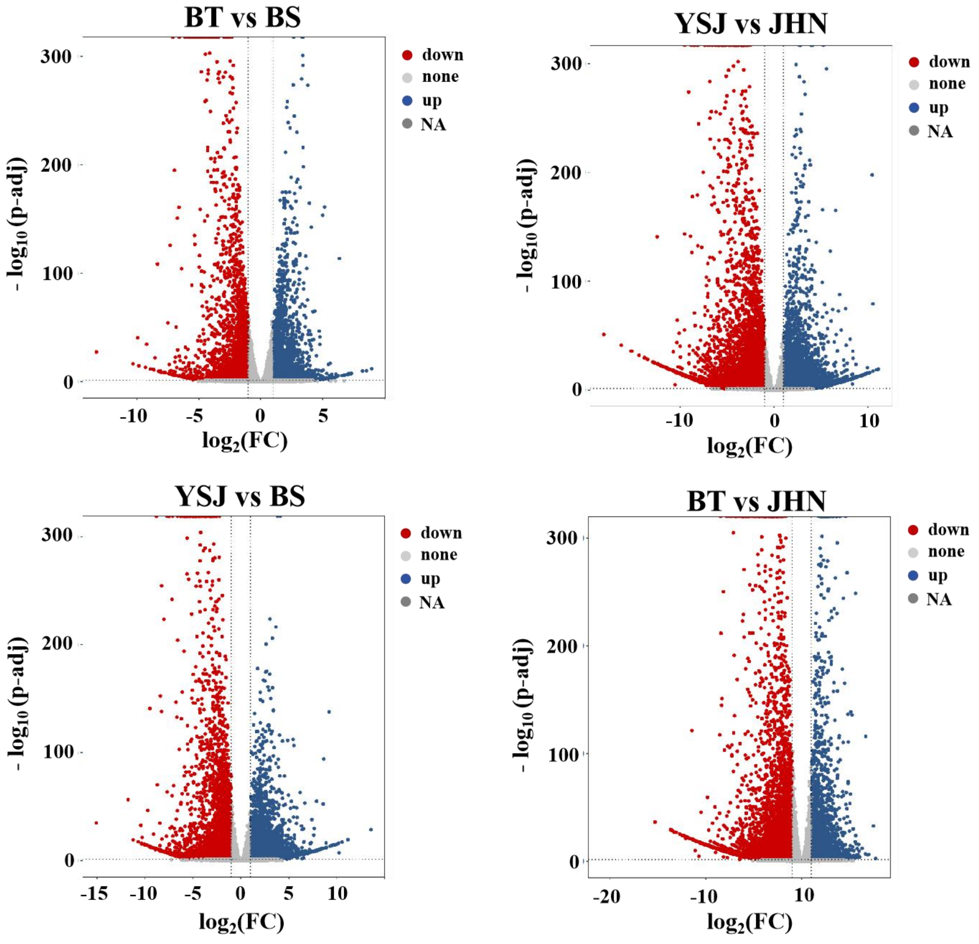

Figure S9 Volcano map of all DEGs in BTvsBS, YSJvsJHN, YSJvsBS and BTvsJHN

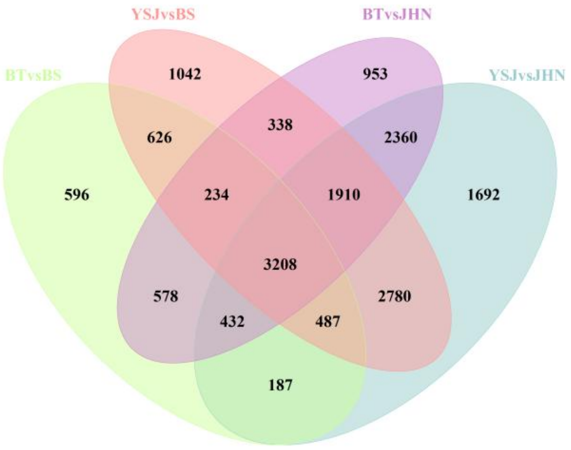

Figure S10 Venn diagram of DEGs in BT vs BS, YSJ vs JHN, YSJ vs BS and BT vs JHN

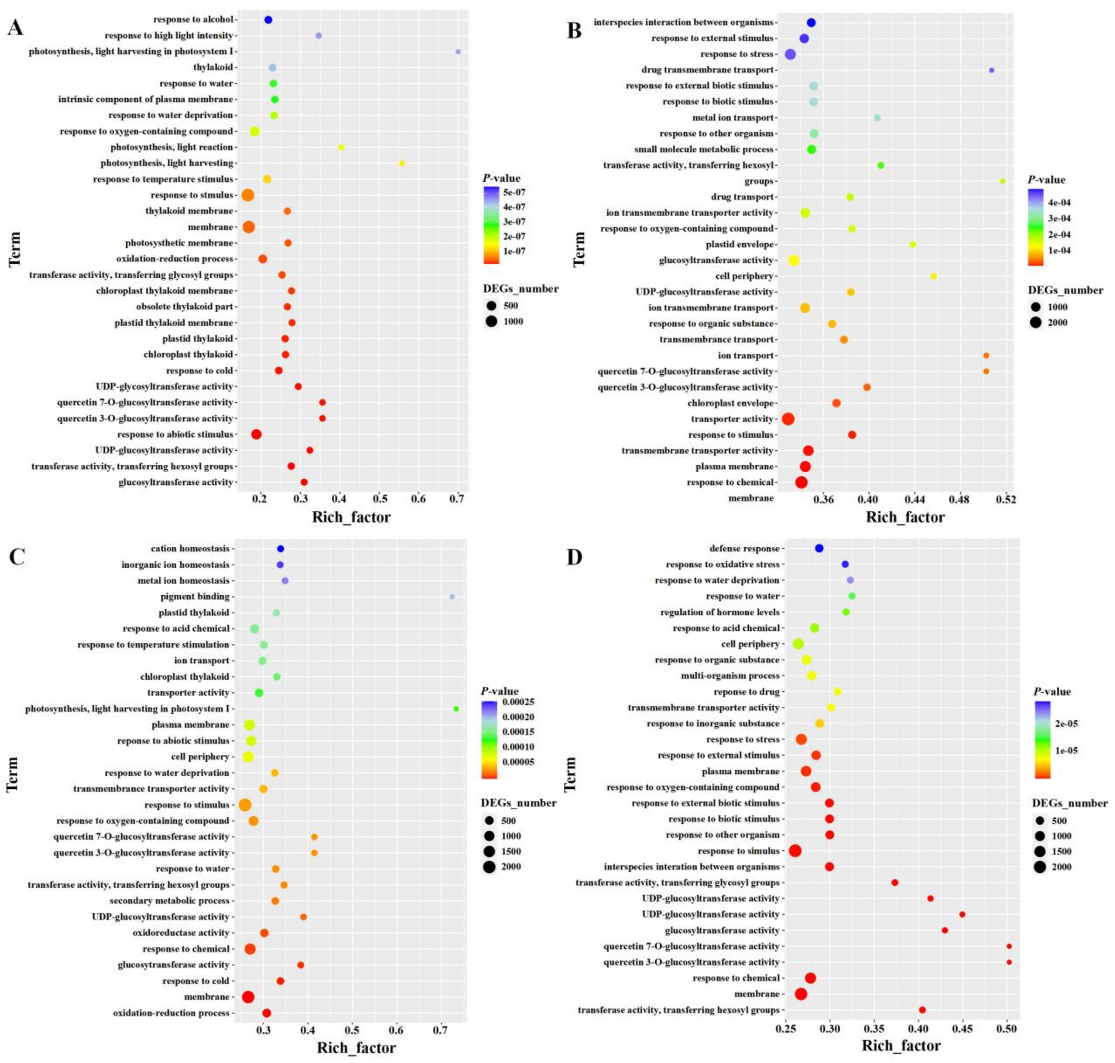

Figure S11 GO annotation of DEGs under drought stress in contrasting groups. A-D represent the BT vs BS, YSJ vs JHN, YSJ vs BS, and BT vs JHN groups, respectively.

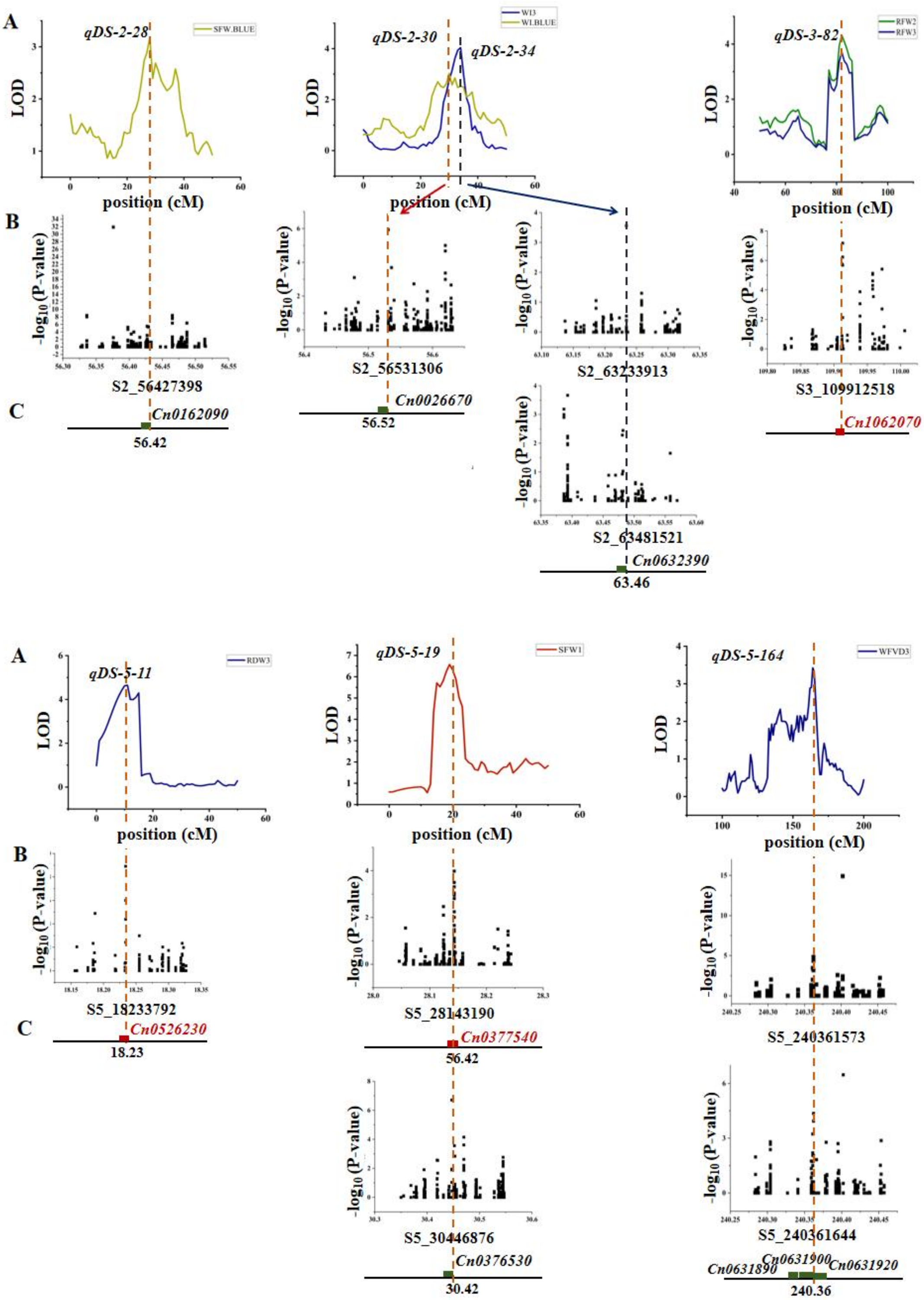
A
B
C
qDS-2-28
SFW.BLUE
qDS-2-30
qDS-2-34
WI3
WI.BLUE
qDS-3-82
RFW2
RFW3
LOD
position (cM)
-log10 (P-value)
S2_56427398
S2_56531306
S2_63233913
S2_63481521
S3_109912518
Cn0162090
56.42
Cn0026670
56.52
Cn0632390
63.46
Cn1062070
qDS-5-11
RDW3
qDS-5-19
SFW1
qDS-5-164
WFVD3
S5_18233792
S5_28143190
S5_30446876
S5_240361573
S5_240361644
Cn0526230
18.23
Cn0377540
56.42
Cn0376530
30.42
Cn0631890
Cn0631900
Cn0631920
240.36

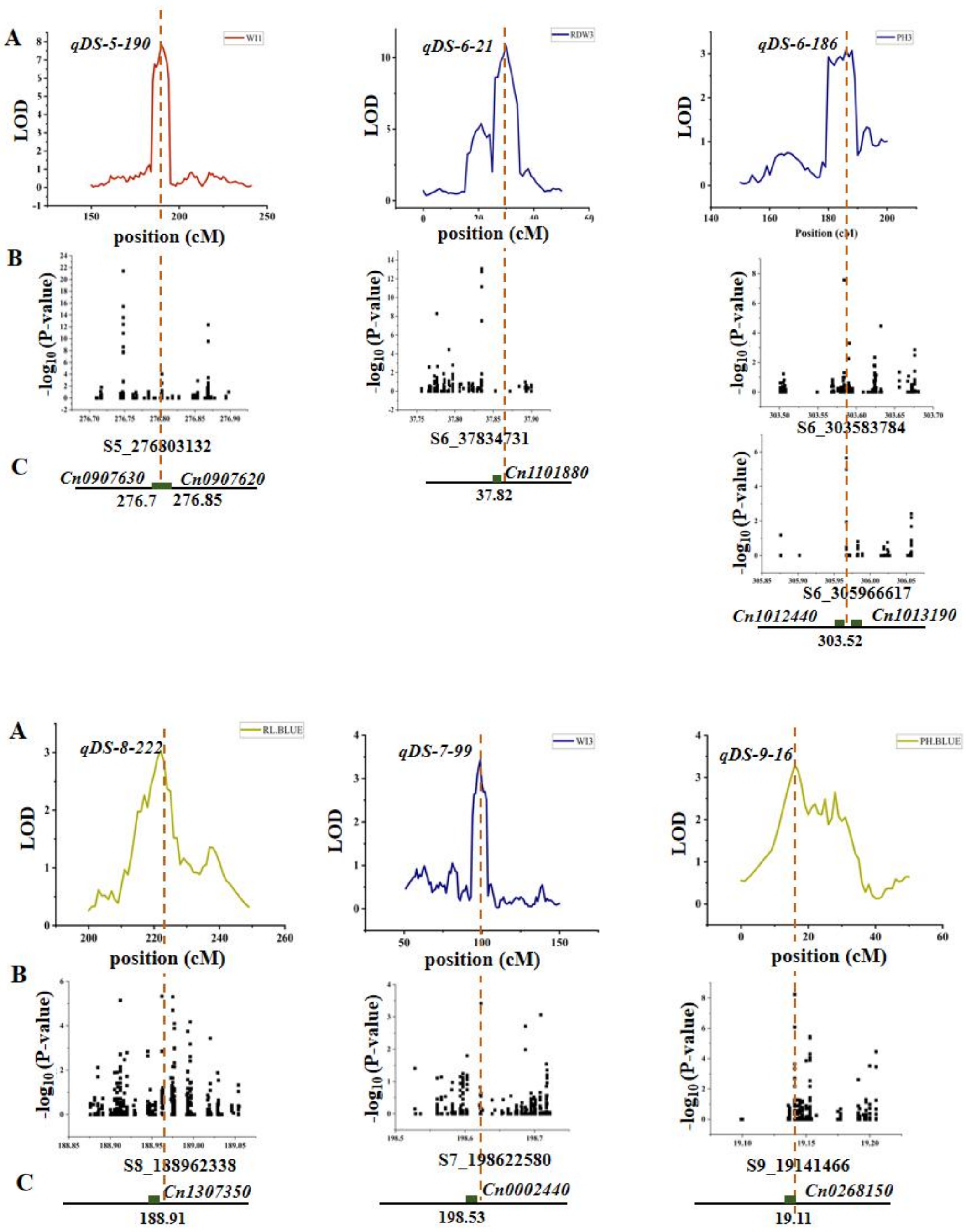

Figure S12 Eighteen candidate genes for drought tolerance identified from the thirty-one QTL-GWAS co-localization loci. (A) LOD curve showing the distribution of significant QTL on the local chromosome region. (B) Manhattan plot of -$\log_{10}$ (*P*-value) for significant SNPs. (C) Schematic diagram of the candidate gene position on the chromosomes. Red regions indicate the genes located in the intergenic regions, while the green regions represent the genes located within 100 kb upstream or downstream of other genes.
